# Fibrotic remodeling and tissue regeneration mechanisms define the therapeutic potential of human muscular progenitors

**DOI:** 10.1002/btm2.10439

**Published:** 2022-11-26

**Authors:** Ya‐Chuan Hsiao, I‐Han Wang, Tsung‐Lin Yang

**Affiliations:** ^1^ Department of Ophthalmology Taipei City Hospital, Zhongxing Branch Taipei Taiwan; ^2^ Department of Ophthalmology College of Medicine, National Yang Ming Chiao Tung University Taipei Taiwan; ^3^ Department of Otolaryngology National Taiwan University Hospital and College of Medicine Taipei Taiwan; ^4^ Research Center for Developmental Biology and Regenerative Medicine, National Taiwan University Taipei Taiwan; ^5^ Graduate Institute of Clinical Medicine, College of Medicine, National Taiwan University Taipei Taiwan

**Keywords:** fibroblasts, fibrosis, matrix metalloproteinase, muscle precursor cells, myofibroblasts, oral submucosal fibrosis, Stem cell

## Abstract

Fibrosis is an intrinsic biological reaction toward the challenges of tissue injury that is implicated in the wound‐healing process. Although it is useful to efficiently mitigate the damage, progression of fibrosis is responsible for the morbidity and mortality occurring in a variety of diseases. Because of lacking effective treatments, there is an emerging need for exploring antifibrotic strategies. Cell therapy based on stem/progenitor cells is regarded as a promising approach for treating fibrotic diseases. Appropriate selection of cellular sources is required for beneficial results. Muscle precursor cells (MPCs) are specialized progenitors harvested from skeletal muscle for conducting muscle regeneration. Whether they are also effective in regulating fibrosis has seldom been explored and merits further investigation. MPCs were successfully harvested from all human samples regardless of demographic backgrounds. The extracellular matrices remodeling was enhanced through the paracrine effects mediated by MPCs. The suppression effects on fibrosis were confirmed in vivo when MPCs were transplanted into the diseased animals with oral submucous fibrosis. The data shown here revealed the potential of MPCs to be employed to simultaneously regulate both processes of fibrosis and tissue regeneration, supporting them as the promising cell candidates for development of the cell therapy for antifibrosis and tissue regeneration.

## 1 INTRODUCTION

Fibrosis originates from the healing process occurring after organ injury. It is associated with excessive accumulation of extracellular matrix (ECM) that may result in remodeling of tissue parenchyma, disorganization of tissue architecture, and potential deterioration of organ function. Fibrosis can be encountered virtually in many different organs, which consequently increases the risk of mortality and morbidity in a variety of diseases.^1^ Although posing major threat in diverse organ systems, fibrosis remains a major clinical challenge worldwide without effective therapies. Therefore, it is imperative to explore the potential therapeutic approaches to suppress development and accelerate regression of fibrosis.

Tissue repair and regeneration is essential for multicellular organisms because of its effect on trauma adaptation and function preservation. These biological activities occur across diverse organ systems by activation and recruitment of tissue‐specific progenitor cells with potency of cell differentiation.^2^ There are common sequential events between regeneration and fibrosis induced by injury. Nonetheless, the outcomes vary as a result of distinct molecular cascades and cellular interactions.^3^ The predisposition is determined by the cellular ability to return to homeostatic status. Alternatively, tissue regeneration is hindered to render the progression shift to fibrosis. Once the point of the bifurcation is reached without further cellular and molecular intervention, the injured tissue may advance to steady state of fibrosis, leaving behind the likelihood of tissue generation and functional recovery.

Stem cells are competent to switch the trend toward the irreversible progression of fibrosis.^3^ If the healing processes of fibrosis can be replaced by regenerative processes, a considerable impact is expected to improve health. When bone marrow derived mesenchymal stem cells (BM‐MSCs) are administered, they serve as the conductor to mediate cellular responses beneficial for tissue regeneration instead of fibrosis.^4^ Nonetheless, BM‐MSCs also have another contradictory role in assisting generation of myofibroblasts (MFs).^5^ These conflicting findings suggest that BM‐MSCs may have dual roles in fibrosis.^6^ On the other hand, pericytes are another cell population residing in a variety of organs with similar MSC features. They participate in injury repair with the ability of tissue regeneration,^7^ but also play double‐edged function to be implicated in the development of fibrosis.^5^ Although with tissue‐specific properties, regulation and determination of the switch between fibrosis and regeneration in pericytes has not been completely understood.^8^ The uncertainty throws a shadow when these cells are selected as the candidates of cell therapy for fibrosis resolution and tissue regeneration.

Since fibrosis is principally driven by the tissue‐specific events occurring in the local microenvironment, the antifibrosis therapy is suggested to focus on tissue‐specific cellular resources.^7^ Muscle precursor cells (MPCs) are the tissue‐specific populations of progenitors reside within muscle, an abundant tissue throughout the whole body. After tissue injury, MPCs re‐elicit cell proliferation, differentiation, migration, and tissue integration to accelerate muscle regeneration.^9^ MPCs have drawn attention because of their promising therapeutic potential. They have been acknowledged for the capacity of tissue repair and regeneration.^10^, ^11^ The outcome of cell therapy confirmes their capacity of promoting functional recovery of injured tissue.^12^, ^13^ Transplantation of MPCs also successfully reconstitute the hematopoietic compartment in the BM, showing their multifunctional potential.^14^ Although with confirmed results to promote tissue regeneration, the role of MPC in regulating fibrosis, which is a critical phase for tissue repair, has seldom been addressed. Regulation of fibrogenic processes is pivotal for tissue repair, and paves way for subsequent tissue regeneration. In the current study, the capacity of MPCs in regulating the tissue‐specific processes of fibrosis and tissue regeneration was investigated and characterized to explore their therapeutic potential of cell therapy.

## 2 RESULTS

### 2.1 Isolation and characterization of MPCs

**FIGURE 1:**
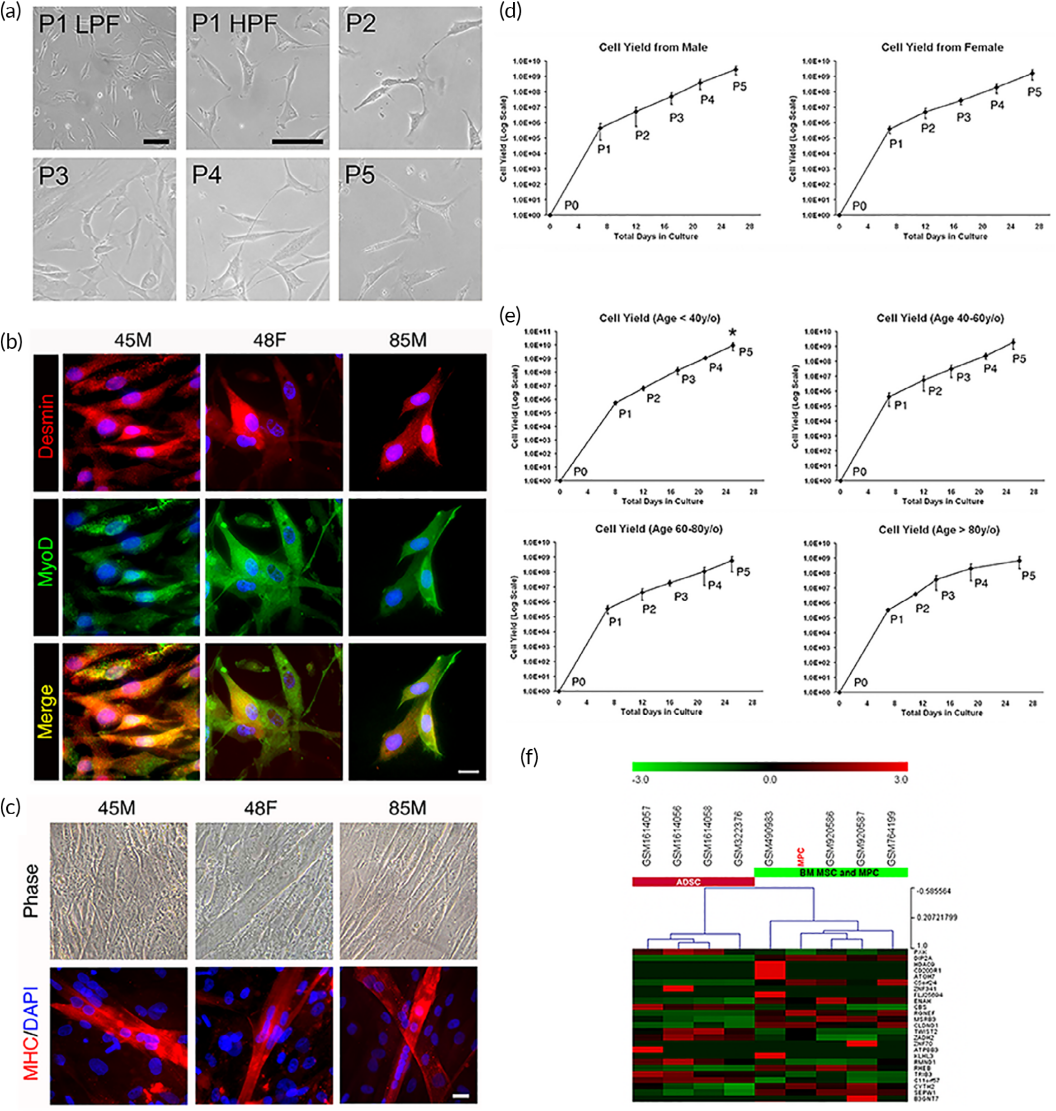
Isolation and characterization of human myogenic precursor cells. (a) Representative phase‐contrast images of the isolated muscle precursor cell (MPC) during passages. Both low‐power field (LPF) and high‐power field (HPF) were demonstrated in P1 while P2–P5 were shown in HPF (scale bar: 50 μm). (b) MPCs harvested from the human subjects with different demographic backgrounds demonstrated the typical markers of MPCs including Desmin and MyoD (scale bar: 20 μm). (c) Muscle differentiation and myotube formation of MPC with different demographic backgrounds. Cultured MPCs were induced to align, fused to become multinucleated, and formed myotubes (upper panel). Myosin heavy chain (MHC) expression was identified in all differentiated myotubes (lower panel; scale bar: 20 μm). Averaged cell yields and expansion potential of MPC from different (d) genders and (e) ages (*n* > 5). (f) A hierarchical clustering Gene Set Enrichment Analysis (GSEA) based on gene expression levels in ADSC, BM‐MSC and MPC, and isolated MPC. Each horizontal row in the figures indicates the gene sets, and the vertical column represents the similar cluster patterns of samples. Red color shows increased fold‐change, black means no difference while green color represents decreased fold‐change. (45M: 45‐year‐old male; 48F: 48‐year‐old female; 85M: 85‐year‐old male; P: passage number; ADSC: adipose derived stem cells; BM‐MSC: bone marrow mesenchymal stem cells)

Human MPCs (hMPCs) were isolated from human donors, and the freshly isolated MPCs demonstrated spindle‐shaped morphology during culture and passage (Figure 1a). The MPCs were confirmed for their characteristics of expressing the markers including desmin and MyoD that were specific to the MPCs (Figure 1b).^9^, ^12^, ^13^, ^15^ These markers were identified in all MPCs harvested from tissue with different demographic background (Figure 1b). Formation of muscle fibers by cell fusion is an important feature of muscle differentiation and maturation. The MPCs were tested for the capacity of myofiber formation when they were induced to differentiate. All MPCs demonstrated cell alignment and fusion for myofiber generation, regardless of the tissue originating from distinct sex or gender (Figure 1c). These myofibers were positively stained for myosin heavy chain, showing muscle maturation of differentiated MPCs (Figure 1c). In addition to characterizing the cellular features of MPCs, the ability of these isolated MPCs to generate sufficient cell numbers efficiently is closely relevant to their therapeutic potential in cell therapy. To evaluate the cellular yield of MPCs, the growth curve was tested up to five passages. All MPC clones grow rapidly especially in the initial P0–P1 state with cell yields averaged to 1 × 10^6^ MPCs in both genders (Figure 1d). Age‐based comparison revealed that only P5 populations with younger‐aged (less than 40‐year‐old) showed significantly higher cell yield compared with relative middle‐aged (40‐ to 60‐year‐old) or older aged (60–80 and older than 80‐year‐old) counterparts (Figure 1e). Within five passages, the cell numbers of all MPCs samples reach 1 × 10^10^, much exceeding the numbers estimated to be required for clinical applications.^16^

Next, we performed molecular profiling of MPC global gene expression to distinguish their similarity with adipose‐derived stem cell (ADSC) and BM‐MSC. Acquisition and analysis of gene expression profiles of other types of stem cells were accessible via Gene Expression Omnibus (GEO) series with accession codes indicated. After comparing these gene profiles, the Gene Set Enrichment Analysis (GSEA) was presented as heat maps with clustering dendrograms (Figure 1f). The data suggested MPC retained muscle features, and expressed the molecular profiling more relevant to BM‐MSC than ADSC. The consistent results were found using gene ontology (GO) term enrichment analysis in search of KEGG and gene symbol database, focusing on signaling transduction and cellular activities individually (Figure S1). The data indicated MPCs isolated and cultured in vitro retained original muscle characteristics while endowed with stem cell properties similar to BM‐MSC.

### 2.2 Regulation of fibrogenesis by MPC

To explore the effect of MPCs on fibrosis, the interaction between MPCs and fibroblasts was investigated. During fibrogenesis, fibroblasts are transformed into MFs and play major roles in the synthesis and deposition of ECM.^17^ In addition to testing the fibroblasts isolated from primary culture of human tissue (hPF), the MFs derived from fibroblasts were also prepared. Identification of MFs is confirmed by the immunophenotypic criteria with the expression of α‐SMA and/or vimentin without desmin.^17^, ^18^, ^19^ They are categorized into different types such as A, V, and VA types depending on the expression of α‐SMA and vimentin.^20^ To avoid undesirable interference caused by cytokine supplements, the cell‐density plating method was employed to prepare MFs in the current experimental approach.^21^, ^22^, ^23^, ^24^ During culture, α‐SMA, a typical marker of MFs, progressively appeared in the cultured cells (day 6) that was not originally detected in the cultured fibroblasts (day 1) (Figure S2a). Quantitative analyses showed significant increases of α‐SMA (Figure S2b), compatible with previous reports.^18^ α‐SMA and vimentin were abundant in the culture with a long period, and persisted after passages (Figure S2b). No differences in the expression of α‐SMA and vimentin were observed between the culture with different cell densities (Figure S2a,b). Once the MFs were identified, reversion of MFs back to fibroblasts were not observed. The data confirmed the success of the current method of inducing the transition from hPFs to MFs that presented as the VA phenotype of MF. Induction of MF formation by transforming growth factor‐beta (TGF‐β) was carried out simultaneously for comparison, which demonstrated the MFs with similar phenotypes^25^ (Figure S2c,d). It had been reported that many intracellular signaling pathways could be activated under TGF‐β treatment during the differentiation process of MFs,^26^ which may result in undesirable biological responses and interaction during the coculture. In addition, TGF‐β might induce the quiescence of myogenic stem cells^27^ Accordingly, the seeding density method that had been well‐recognized as the standard method to prepare MFs was employed to avoid the interference of exogenous growth factors by reagent supplement.

**FIGURE 2:**
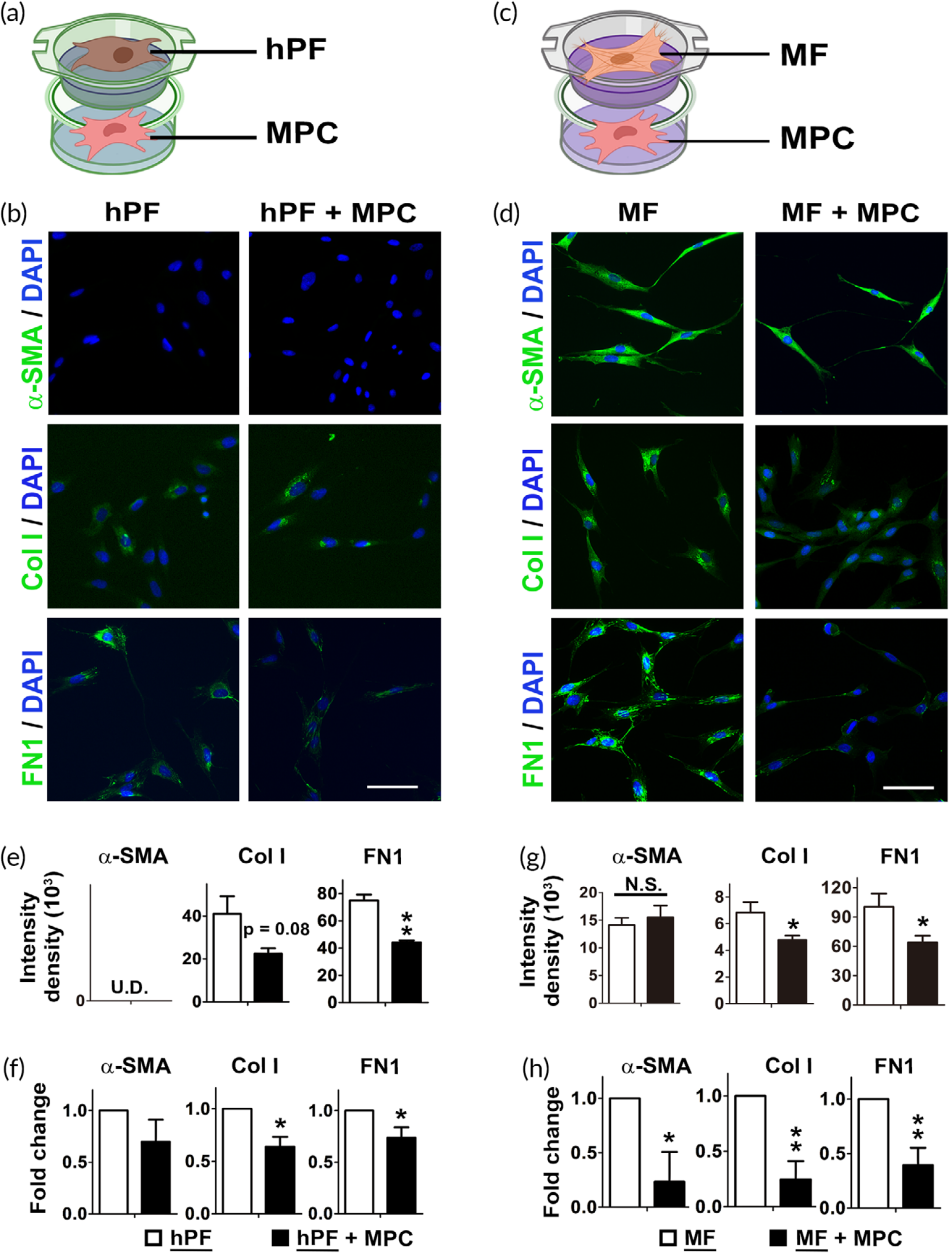
The effect of muscle precursor cell (MPC) coculture with hPF or MF on fibrosis‐associated markers. The illustration shows the coculture systems of (a) hPF, or (c) MF with MPCs by trans‐well experiments. Immunofluorescent staining of α‐SMA, Col I, and FN1 (green) was performed to evaluate the expression of fibrosis‐associated markers in (b) hPFs and (d) MFs. (Nuclei: blue; scale bar: 50 μm). Quantification of the fluorescent intensity of α‐SMA, Col I, and FN1 was presented in (e) hPFs and (g) MFs with or without MPC coculture. Quantitative results of real‐time PCR of these makers in (f) hPF and (h) MF with or without MPC coculture. (hPF: human primary cultured fibroblasts; MF: myofibroblasts; α‐SMA: α‐smooth muscle actin; FN1: fibronectin; Col I: type I collagen; underline: the targets to be compared; *n* ≥ 3 biological replicates).

The coculture systems were prepared to explore the effects of MPC on hPF or MF. The trans‐well experiments offering non‐contacting cells that showcase only paracrine signaling was prepared. As schematic depiction, an insert coculture system was accomplished by seeding MPCs in the lower compartments while hPF or MF were placed in the upper inserts (Figure 2a,c). To further elucidate MPC effect on fibrinogenesis, expression of ECM markers in hPF and MF was investigated. IF showed that cocultures of MPCs resulted in generally lower expression of collagen type I (Col I) and fibronectin 1 (FN1) in hPFs (Figure 2b) or MFs (Figure 2d). Quantification of the relative values of fluorescent intensity confirmed the results (Figure 2e,g). FN1 is an important marker of MF phenotype.^28^ The high level of FN in MF was reduced when MPC was present. Real‐time PCR was performed to further confirm the results; MPC demonstrated a consistent trend of reducing fibrosis‐associated markers in hPFs (Figure 2f). The effect was greater when MF was tested rather than hPF (Figure 2h). Consistent with the findings, the results of flow cytometry revealed that the numbers of the cells carrying the specific markers of hPF or MF were reduced by MPC (Figure S3).

In addition to examining the change of hPF and MF during coculture, alteration of cellular properties of MPC caused by the reciprocal interaction was investigated. It provided evidence to verify whether coculture interferes with the original phenotypes of MPC. When the typical markers of muscle precursors were checked, MPCs still preserve the primitive features, showing the patterns compatible with muscle precursors after MF coculture (Figure S4a). Since the progenitor cells are reported to be probably differentiated into MF during fibrogenesis, the possibility was further examined by the expression of typical fibrotic markers in MPC after coculture. Compatible with the data mentioned above, the induced MF belonged to VA types with the positive expression of α‐SMA and vimentin (Figure S4b).^17^ Nonetheless, no expression of α‐SMA was detected in MPC regardless of being cocultured with hPF or MF (Figure S4b). In addition, expression of vimentin was originally detected in MPC. When MPC was coculture with hPF or MF, its expression decreased (Figure S4c). The data suggested that the original phenotypes of MPCs were not largely changed by coculture, and the possibility of the differentiation toward MF was not observed as well.

**FIGURE 3:**
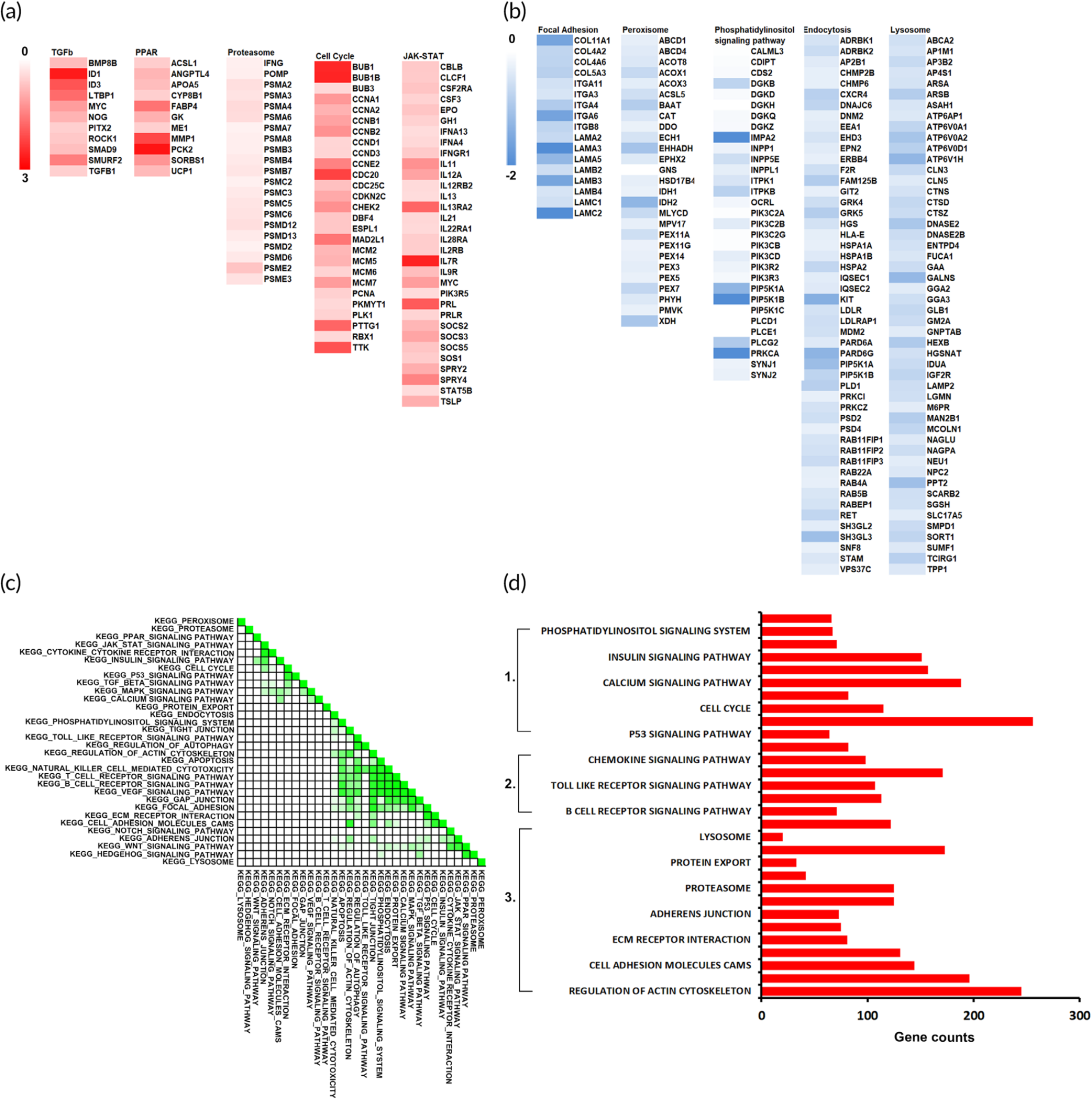
Extracellular matrix (ECM) regulation comprises gene expression signature in the human primarily cultured fibroblast (hPF) cells cocultured with muscle precursor cell (MPC). (a, b) Gene Set Enrichment Analysis (GSEA) pre‐ranked analyses of leading‐edge genes were indicated. The comparison was performed by the definition as the levels in hPF culture with or without MPC. The figure presents (a) upregulated genes and (b) downregulated genes. (c) GSEA set‐to‐set analysis shows the enrichment of markers implicated in ECM remodeling. (d) Gene ontology analysis for the enrichment was grouped into three clusters: 1. cell signaling, 2. immune cell‐related, 3. protein degradation and ECM remodeling.

Next, we took advantage of a transcriptomic study on hPFs to investigate whether the differences of ECM remodeling were present in the culture with MPC. GSEA leading‐edge analyses of the genes upregulated (Figure 3a) or downregulated (Figure 3b) in hPFs culture with or without MPCs were performed. It demonstrated that the proteasome represented the top‐ranked categories in the upregulated edges, whereas ECM and focal adhesion pathway represented the top‐ranked categories in the downregulated edges. In the leading‐edge analysis, matrix metalloproteinase 1 (MMP1) was found to be upregulated (Figure 3a). On the other hand, downregulation of integrins, collagens, and laminins were identified (Figure 3b). These results confirmed that MPCs increased the activities of protein degradation and reduced the expression of fibrosis‐associated markers. Accordingly, genes encoding focal adhesion, ECM receptor interaction, and adherent junction were highly emphasized in GESA set‐to‐set KEGG analysis (Figure 3c). To further verify the significance of transcriptomic profiling, we performed GO analysis in the gene enrichment of hPFs with or without MPC coculture. The comparative analysis identified main categories associated with protein degradation and ECM remodeling, including proteasome, adhesion molecules, and cytoskeleton (Figure 3d). Altogether, these data demonstrated that MPCs preferentially downregulated fibrosis‐associated genes, and promoted functional networks and interaction implicated in ECM degradation and remodeling.

### 2.3 Fibroblast/MFs cocultured with MPC increased the proteolytic activities of ECM through MMPs

**FIGURE 4:**
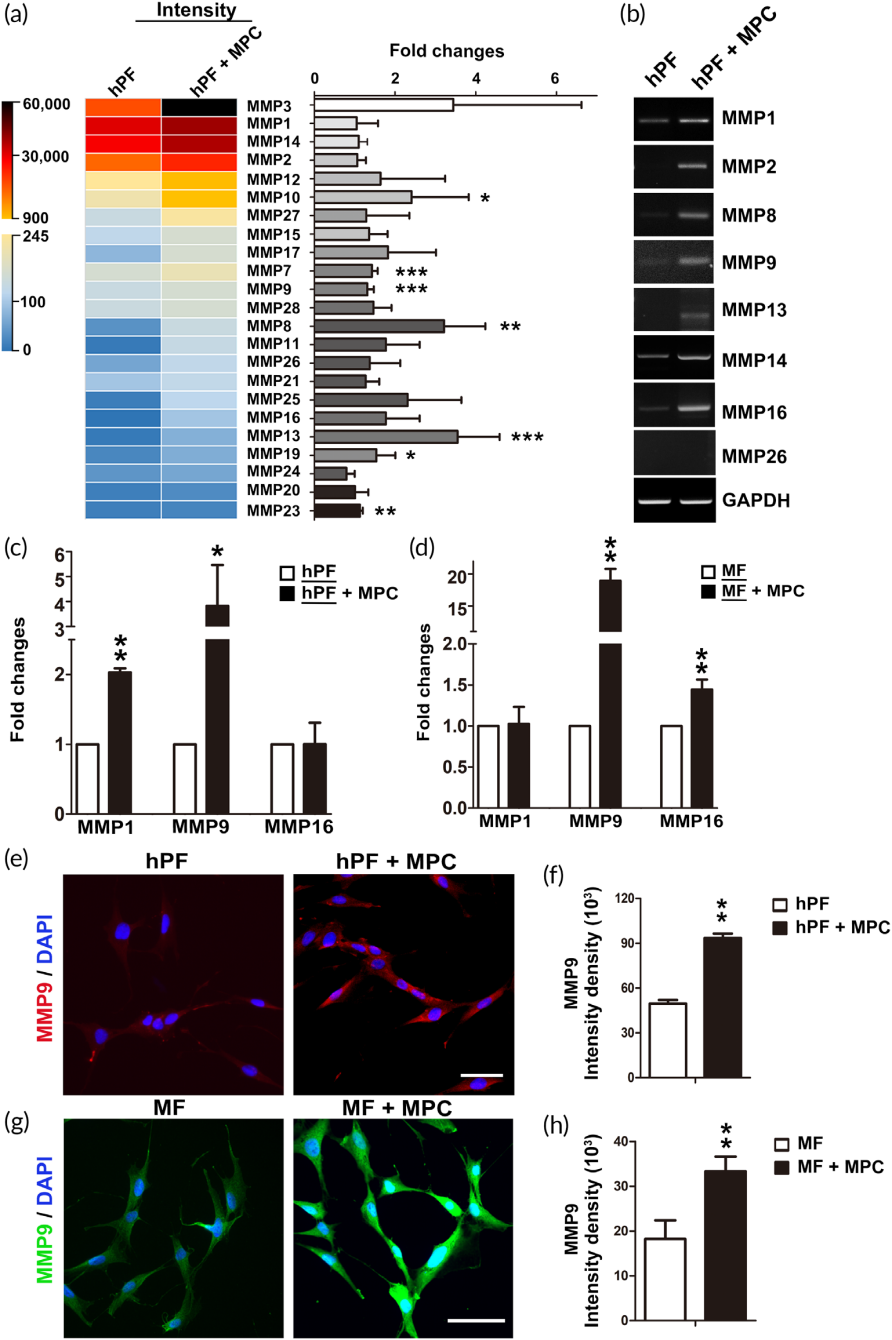
Effects of muscle precursor cell (MPC) on matrix metalloproteinase (MMP) produced by fibroblasts/myofibroblasts. (a) The color‐coded heat map demonstrated the differential elevation intensity of MMPs in the fibroblasts cocultured with or without MPCs. The bar chart indicated the fold changes of MMPs. Gene expressions of MMP1, 2, 8, 9, 13, 14, 16, and 26 in human primarily cultured fibroblasts (hPFs) cultured with or without MPCs were assayed by (b) RT‐PCR, and (c, d) real‐time PCR. (e) The hPF and (g) MF were stained for MMP9. (scale bar: 50 μm). Quantification of the relative fluorescence integrated density of MMP9 in (f) hPF and (h) MF (*n* ≥ 3 biological replicates).

ECM provides structural support and dynamic interaction that reciprocally influence the biological behavior of dwelled cells. Remodeling of ECM is a delicate process that requires homeostasis and is exerted by physiological or chemical modification. The MMP family, a special family of zinc‐dependent endopeptidases, plays a central role in ECM degradation and remodeling.^29^ downregulated expression of MMPs has been reported as an important mechanism in fibrogenesis.^30^ Accordingly, we hypothesized that coculture of MPCs may stimulate the expression of MMP family, subsequently leading to ECM degradation. The expression level of MMP family in the fibroblasts cultured with or without MPC was determined by transcriptomic approach. The results were demonstrated as intensity heat maps (Figure 4a, left) and fold changes (Figure 4a, right). The majority of MMP family differentially increased gene expression in the coculture. PCR gel electrophoresis showed increased expression of the MMP family as well (Figure 4b). There are several subgroups of the MMP family, including collagenases, gelatinases, and membrane‐type MMP. MMP1, MMP9, and MMP16 are the representative of each subgroup, respectively.^31^ Their expression in the hPF or MF culture with or without MPC were examined by real‐time PCR. It was found the expression of MMP1, MMP9, and MMP16 significantly increased when hPFs (Figure 4c) or MFs (Figure 4d) were cocultured with MPCs. The hPFs cocultured with MPCs expressed significantly elevated MMP9 as demonstrated in the IF images (Figure 4e) and the quantification of intensity density (Figure 4f). Similar to hPFs, MPC also affected the expression of MMP9 expression in MFs (Figure 4g,h).

**FIGURE 5:**
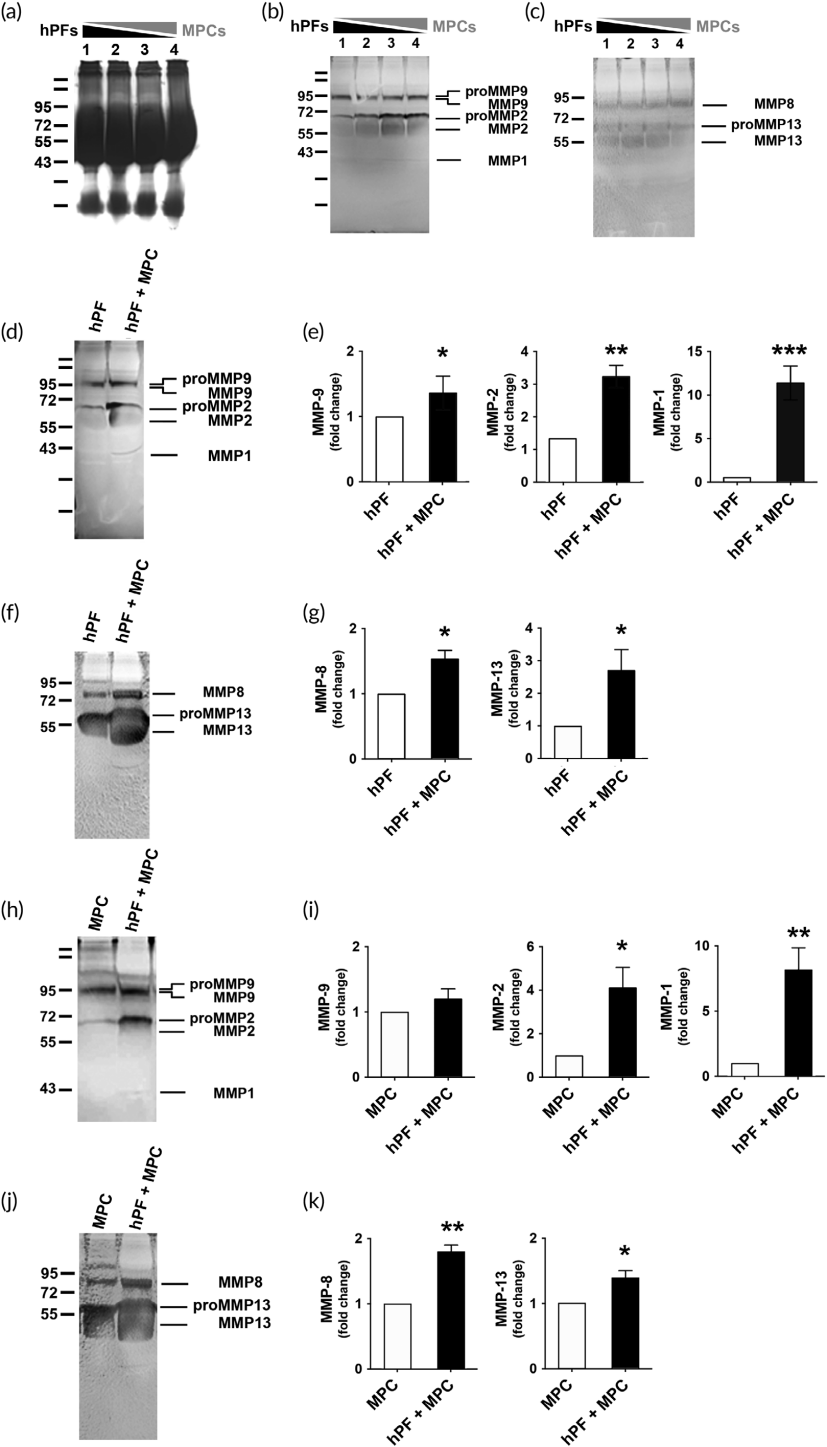
Coculture of muscle precursor cell (MPC) with fibroblasts increased matrix metalloproteinase (MMP) activities. (a) Coomassie‐stained gels showed equal amount of total loading in each lane. Collagen/gelatin in gel zymography analyses with the titration of human primarily cultured fibroblasts (hPFs) by MPCs demonstrated dose‐dependently activation and increased activities of (b) MMP1, MMP2, MMP9, and (c) MMP8, MMP13. With MPC, MMP activation and substrate degradation increased in hPF for (d) MMP1, MMP2, MMP9, and (f) MMP8, MMP13, with quantitative analyses normalized to hPF (e, g). With hPF, MMP activation and substrate degradation increased in MPC for (h) MMP1, MMP2, MMP9, and (j) MMP8, MMP13, with quantitative analyses normalized to MPC (i, k) (*n* ≥ 3 biological replicates).

Accordingly, we wondered whether MPCs would facilitate the proteolysis of ECM components by secreting and activating MMPs. Activated MMPs degrade collagen in ECM.^32^ Therefore, collagen/gelatin zymography was employed to provide the direct evidence. Before sample loading, the equal amount of each group was ensured (Figure 5a). In the in gel zymography of coculture, activation and activities of MMP1, MMP2, MMP8, MMP9, and MMP13 increased. MMP1, MMP8, and MMP13 are interstitial collagenases with the function of degrading type I and III collagen, while MMP2 and MMP9 are categorized as gelatinases to exert proteolysis of gelatin, type IV collagen, and others.^33^ These ECM substrates are essential components of fibrosis. Titration of hPFs by MPCs demonstrated dose‐dependent manner in enzyme activation and substrate degradation (Figure 5b,c). The results showed remarkable upregulation of the active forms and degradation abilities of MMPs in the coculture of MPC and hPF. To further verify whether the effect of coculture originated from hPF or MPC, these cells were analyzed individually. When hPF was cultured with MPC, the expression of cleavage bands in the zymography of MMP1, MMP2, MMP9, MMP8, and MMP13 increased when compared with the control of hPF only (Figures 5d–g). In a parallel study, similar results were found in the coculture of MPC and hPF when the data were normalized to the control group with MPCs only (Figure 5h–k). The biochemical data indicated that enzyme activation and substrate degradation efficacy of MMPs were enhanced in the coculture of fibroblasts and MPCs.

To further explore the effects of MPCs that dictated on matrix remodeling, the expression levels of TGF‐β and the tissue inhibitors of metalloproteinases (TIMPs) were investigated. Bioactive TGF‐β has been confirmed to promote the pathogenic fibrotic responses and participate in creating a profibrogenic microenvironment.^34^ TIMPs are natural efficient inhibitors of many MMPs to regulate proteolysis.^35^ The TGFβ1, TGFβR1, TGFβR2, TIMP1, and TIMP2 gene expressions were examined through qPCR in hPF and MF. Results showed that MPC coculture generally reduced TGF‐β family expression together with TIMPs levels (Figure S5). Taken together, the data suggest that interactions of hPF or MF with MPC result in increased MMP activation and activities, and subsequently facilitating ECM remodeling.

**FIGURE 6:**
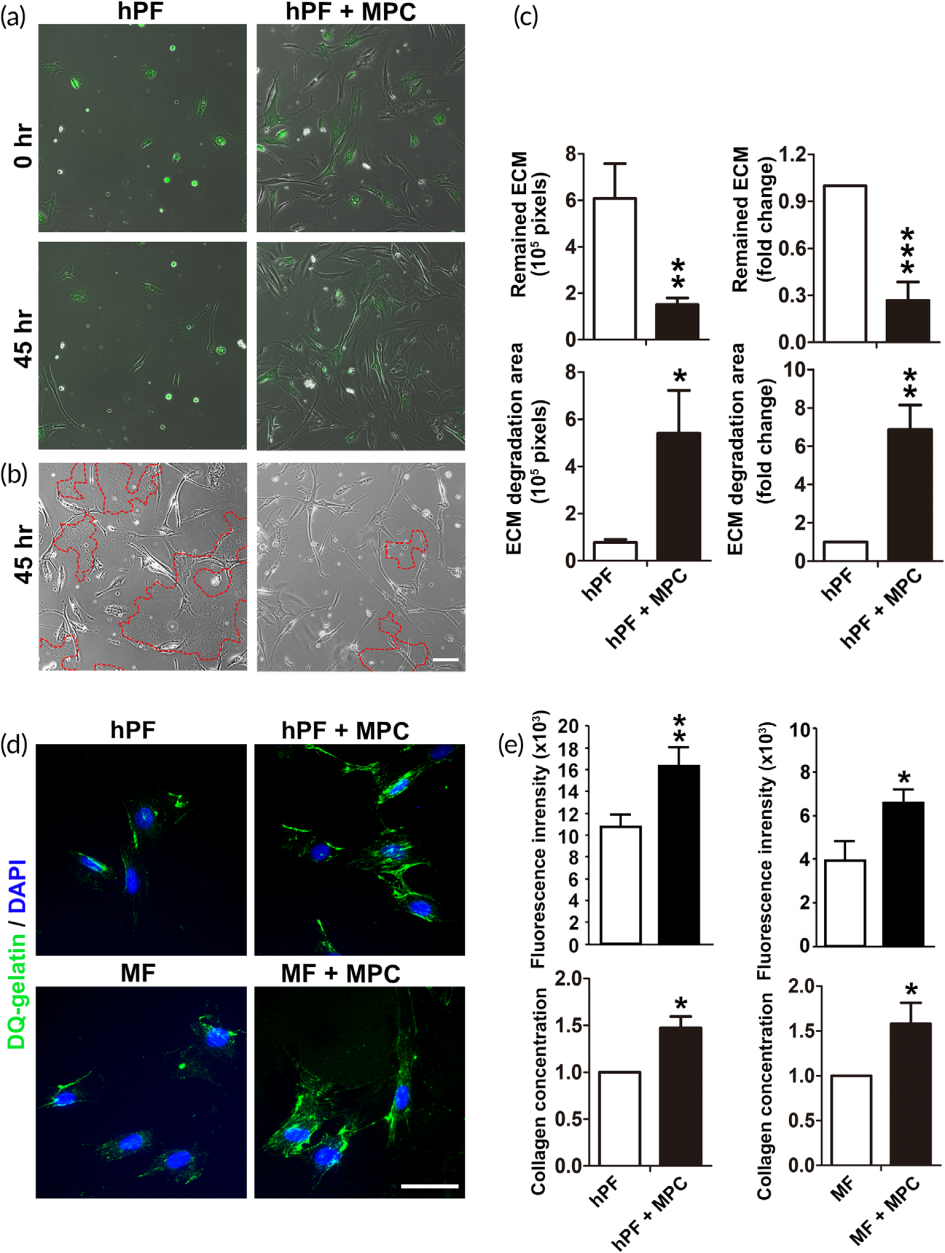
Increased extracellular matrix (ECM) degradation by cocultured fibroblasts with muscle precursor cell (MPC). (a) The fluorescence‐labeled human primarily cultured fibroblast (hPF) upon culture with or without MPC on collagen at 0 and 45 h. (b) The clearance area represented collagen degradation in both groups. The red dashes encircled the remaining collagen. Scale bars: 100 μm. (c) Quantification of the remained and degraded ECM in hPF culture with or without MPC. (d) Degradation of DQ‐gelatin (green fluorescence) in the cocultures of MPC with hPF (upper panel) or myofibroblast (MF) (lower panel). Scale bar: 50 μm. (e) The fluorescent intensities and collagen concentrations were measured and compared (*n* ≥ 3 biological replicates).

To confirm the ability of ECM degradation directly, hPFs were cultured on the collagen‐coated environment with or without MPC. The phenotypes of hPFs were observed during coculture with MPCs using fluorescent labeling. The cocultured hPFs maintained slender‐shaped morphology throughout the whole culture period (Figure 6a). It supports the observation that bidirectional communication between ECM and resident cells affects not only the surrounding matrix but also the cell phenotypes.^36^ The clearance area of ECM degradation was measured in both groups. The quantitative results showed that the area of remained ECM was significantly reduced while in accordance with the elevation of ECM degradation area in the coculture (Figure 6c). To further verify the extent of ECM degradation, in situ zymography was employed to test the MPC effect by measuring the ECM degradation area of hPFs with or without coculture (Figure 6d). Fluorogenic DQ‐gelatin/collagen has been approved for the sensitivity of measuring the gelatinolytic activity^37^ and was accordingly employed in the coculture. The fluorescent signals developed upon occurrence of proteolytic events. The results of fluorescent images showed that the proteolytic ability elevated in hPFs in the coculture with MPCs (Figure 6d,e). Consistently, MPCs increased the levels of DQ‐gelatin cleavage in MF coculture (Figure 6d,e). The effect might be challenged to be caused by proliferation of hPFs/MFs triggered by MPC, therefore, the proliferative assays using Ki67 were performed. The co‐expressed KI67 levels in hPF or MF with MPC cocultures showed similar staining patterns compared with those without coculture, suggesting the effect of MPCs on hPF on MF was not toward proliferation (Figure S6). These data provided evidence in the cellular levels to confirm the effect of MPC coculture in facilitating ECM degradation.

### 2.4 Alteration of secretome in the coculture of fibroblasts/MFs with MPCs

**FIGURE 7:**
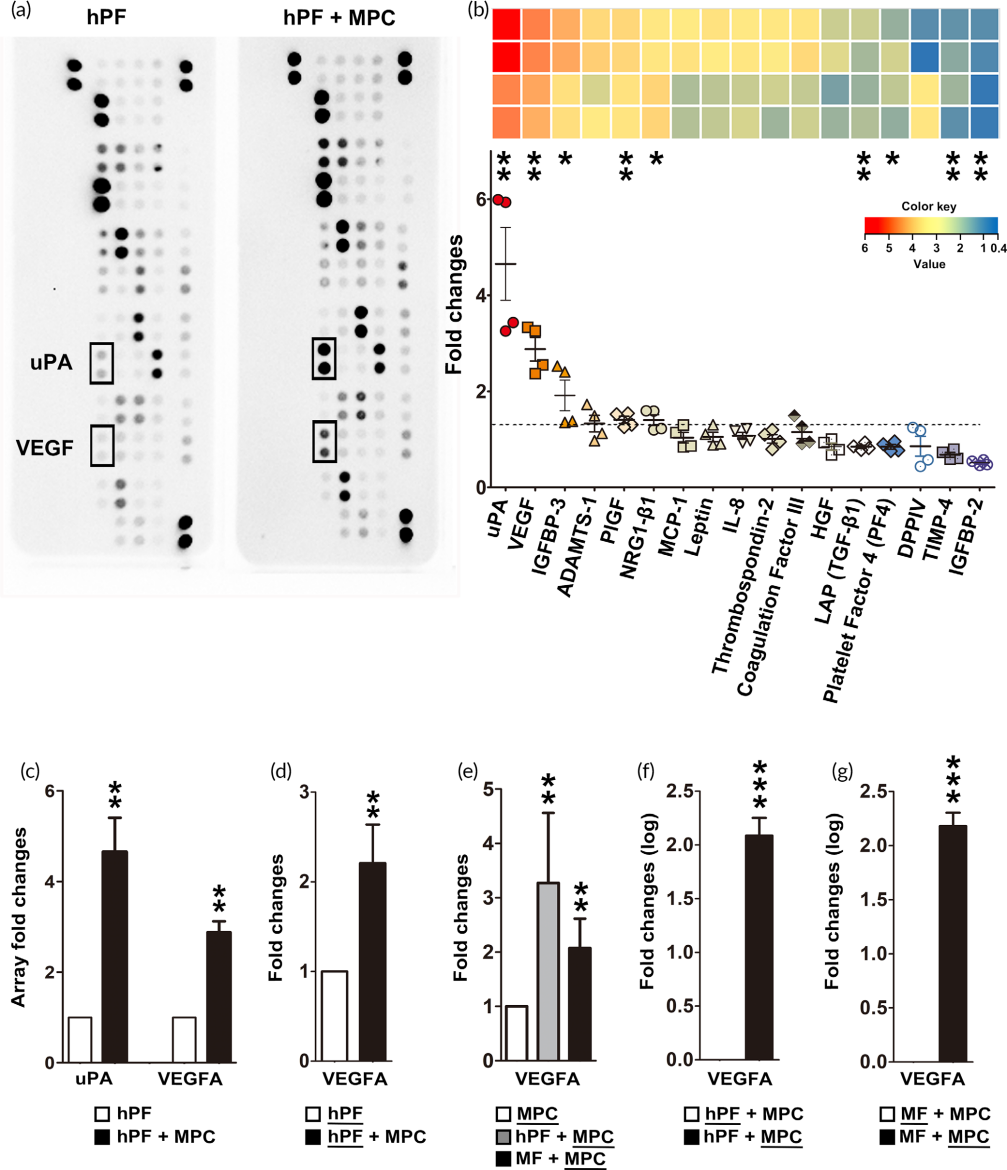
Secretion of urokinase‐type plasminogen activator (uPA) and vascular endothelial growth factor (VEGF) was enhanced by muscle precursor cell (MPC) coculture. (a) The Proteomic Array was used to assess the protein levels of conditioned media from human primarily cultured fibroblast (hPF) culture and those with MPC coculture. (b) A dot plot showing the selective quantification of the fold changes in protein levels. Each row of heat map corresponds to an individual gene in the dot plot. (c) Quantification of the microarray fold changes in uPA and VEGF. (d–g) Real‐time PCR analysis of VEGFA expression levels. The cells used for comparison were underlined (*n* ≥ 3 biological replicates).

The stem cells play critical roles in ECM remodeling by modulating the secretion of bioactive molecules.^38^ According to the results mentioned above, we have confirmed there is a close connection between MPCs and ECM modification. However, the microenvironment of protein networks responsible for MPC effects remained elusive. To this end, the protein array encompassing essential cytokines and growth factors were analyzed in four‐time repeats to compare the profiles of the conditioned media from hPF culture, with or without MPC (Figure S7). Results of the secretory protein blots on conditioned media collected from the coculture of hPF with MPCs showed increased signals of urokinase‐type plasminogen activator (uPA) and vascular endothelial growth factor (VEGF) (Figure 7a). The measurements were normalized to the profiles of hPF groups, and the fold‐changes were represented as a heat map (Figure 7b, upper). For these proteins, the data from the highest to the lowest value in the distribution were further calculated for significance (Figure 7b, lower). Quantification of the two secretory proteins, uPA, and VEGF, were significantly overexpressed in the MPC cocultures compared to the hPF‐alone controls (Figure 7c). To confirm the cellular origins of VEGF, real‐time PCR was performed for the cultures of hPFs of MFs, with or without MPCs. The results showed that VEGF expression followed the trend demonstrated in the protein array. VEGF was found to be more highly expressed in hPF when MPCs were cocultured than in the hPF control group (Figure 7d). Intriguingly, MPC secrets more VEGF in the coculture system than culture alone (Figure 7e). In the comparison of the secretion levels of indicated cellular origins, VEGF was found to be much higher in MPCs than in hPFs (Figure 7f), or MFs (Figure 7g) during coculture. The effects of enzymatic degradation mediated by VEGF signaling in MPCs were further investigated. The DQ‐gelatin degradation assay and the immunofluorescence staining of MMP9 were applied in the hPFs/MFs and MPC cocultures after treatment of VEGF recombinant protein or neutralizing VEGF antibody. VEGF promoted the gelatin degradation effects in the coculture with MPC and upregulated the MMP9 expressions, either in hPF or MF. When VEGF neutralizing antibody was used, the degradation effects were suppressed in the coculture with MPC and the MMP9 expressions decreased, both in hPF and MF (Figure S8). When compared with hPF/MF single‐cultures, the cocultures with MPC still presented higher degradation ability and MMP9 levels, even under the treatment of VEGF‐neutralizing antibody (Figure S8). These experiments show consistent results in the hPF/MF when cocultured with MPC, confirming the role of VEGF in the effect mediated by MPCs on hPF/MF. The results indicate that MPC is principally responsible for VEGF secretion. Both MPC and fibroblasts/MFs increased VEGF secretion during coculture contribute to the mutual interaction between these cells for tissue remodeling.

### 2.5 Transplantation of MPC in the animal model of oral submucous fibrosis

**FIGURE 8:**
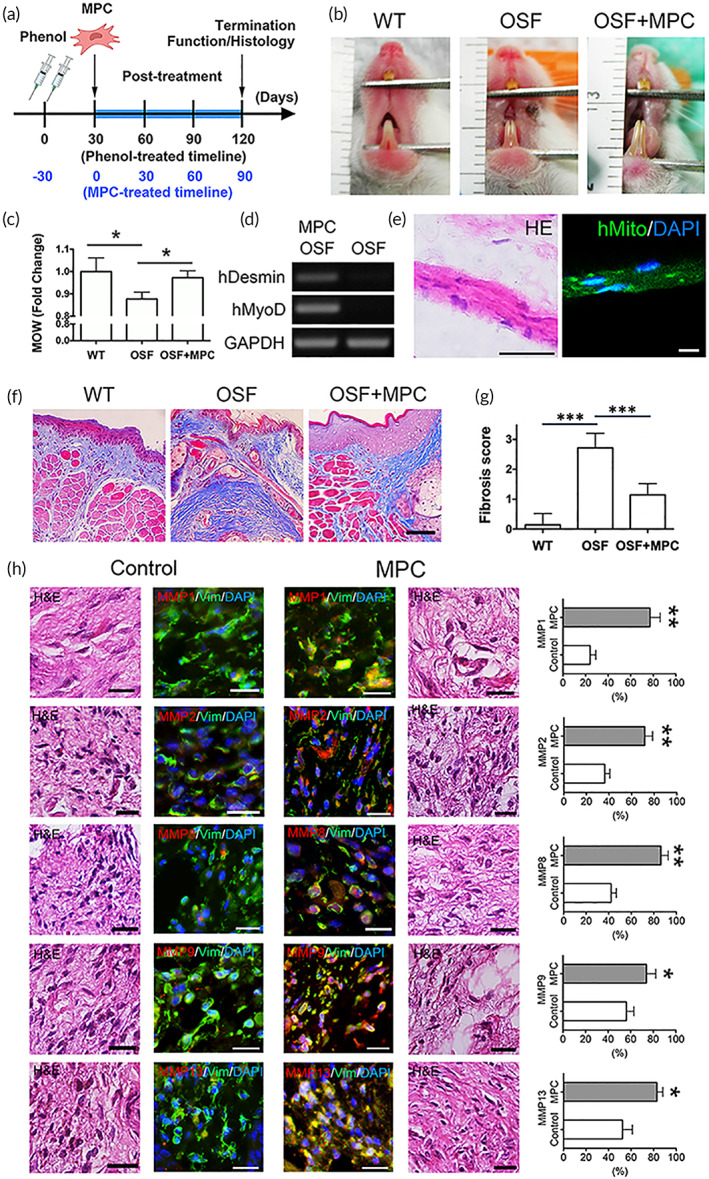
The in vivo function of muscle precursor cells (MPCs) in the animal model of oral submucous fibrosis (OSF). (a) A schematic of the experimental schedule. Phenol was initially injected into the oral cavity to induce OSF. After 30 days, MPCs were transplanted in vivo. (b) Representative images of the open state of mouth in each group. (c) Quantification of the mouth opening width among these groups. (d) mRNA expression of human muscle‐specific intermediate filament desmin (hDesmin) and human myogenic regulator MyoD (hMyoD) in the submucosa layer of MPC‐treated OSF mice and the controls. (e) The muscle fibers with the positive staining of human mitochondria in the submucosa of the MPC treated groups. (f) Evaluation of collagen deposition (blue) in the submucosal layer using Masson's trichrome staining. (g) The degree of fibrosis was scored in the experimental and control groups. (h) Representative H&E and immunofluorescent staining of matrix metalloproteinase 1 (MMP1), MMP2, MMP8, MMP9, MMP13 (red) and vimentin (green) in the control (OSF) and MPC (OSF + MPC) groups. Fluorescent density was quantitatively compared (WT: wild type; MOW: mouth opening width; *n* ≥ 3 biological replicates).

MPC has long been recognized for its regenerative capacity.^10^, ^11^ Transplantation of MPC into damaged sites has been documented to recover physiological function.^12^, ^13^ Given the promise of MPC for disease treatments, their influence on fibrogenesis in vivo merits further investigation. The effect of MPC was examined in the animal disease model of oral submucous fibrosis (OSF) induced by phenol.^39^ The cumulative phenol exposure leads to OSF that results in limited mouth opening. MPC was transplanted and evaluated for in vivo effect as scheduled (Figure 8a). In the group treated with MPC, limitation of mouth opening improved (Figure 8b). The mouth opening width in the OSF animals recovered to the same degree as those of the untreated wild‐type group (Figure 8c). To confirm the existence and survival of MPCs when transplanted in vivo, the tissue specimens were harvested after the experiments. Expression of human desmin and MyoD gene was observed, which verified the existence of MPC during the period of in vivo experiment (Figure 8d). The presence of muscle fiber with the immunofluorescent staining of human mitochondria in the submucosa of the MPC treated groups confirmed cell survival and tissue regeneration by MPC (Figure 8e). Assessment of collagen fiber accumulation revealed restricted area of collagen deposition and morphologically re‐delineation of the mucosa and submucosa layers in the MPC treatment group (Figure 8f). The degree of fibrosis decreased in the group of MPC treatment (Figure 8g). Gross evaluation of MMP expressions in tissue sections demonstrated that the MPC‐treated groups had significantly higher levels of MMP expression than those of the control counterparts (Figure 8h). In summary, the data demonstrated that MPCs were competent to survive, differentiate, and interact with microenvironment after transplantation. Tissue remodeling mediated by MPCs in regulating MMPs and facilitating tissue regeneration is beneficial for fibrosis alleviation in vivo.

## 3 DISCUSSION

MPCs demonstrated the capacity to simultaneously possess dual functions of facilitating fibrosis resolution and tissue regeneration, which constitute the major parts of the scenarios for tissue repair. The analyses of molecular signature and cellular phenotypes confirmed that MPCs had the same features as satellite cells, but not for those of pericytes.^8^ Therefore, MPCs may take advantage of tissue‐specific properties to promote tissue regeneration and be avoid the disadvantages of the features relevant to the embryonic mesoangioblasts that have double‐edged effects in fibrosis progression.^8^ During tissue healing and regeneration, growth factors and cytokines are potent mediators supporting the essential biological events. However, their consistent and precise spatiotemporal release is difficult to maintain. It is therefore cells are used instead as the delivering vehicles to propel the advancement of cell therapy. Nonetheless, appropriate determination of the cellular sources that possess the preferential features without adverse effects is critical for the success of tissue repair. Cells are basically phenotypic and functional dynamic, particularly for the progenitors with multi‐potency. For the success of cell therapy, the expected cellular functions should be augmented, whereas the unexpected drawbacks need to be inhibited. MPCs simultaneously regulate the activities and transition of fibroblasts to suppress fibrosis progression, participate in ECM degradation, and differentiate into mature muscle fiber, which altogether have promoting effects on tissue regeneration (Figure S9).

The interaction between MPCs and fibroblasts is beneficial for tissue regeneration. It had been reported that the positive crosstalk between these cells promotes tissue regeneration in the early stage of tissue repair and suggested that the reciprocal interaction by myogenic cells may negatively regulate fibroblasts to inhibit excessive fibrosis.^40^ During the early stage of regeneration, satellite cells differentiate into myofibers together with self‐renew. Replenishment of the pool of satellites cells depends on the positive feedback of the interaction between satellite cells and fibroblasts.^40^ In the later stage, it is hypothesized that fibroblasts are negatively regulated to avoid excessive fibrosis. Although the interaction of MPCs and fibroblasts in the early stage has been studied, the latter part related to suppression of the progression of fibrogenesis has seldom been addressed. The current study confirmed the hypothesis for the first time, and explained the mechanism to support the cellular effects of MPCs in regulating fibrosis. Our data demonstrated that MPC potently maintained the original phenotypes of fibroblasts, suppressed the transition from fibroblasts to MFs, and preserved the primitive myogenic features without showing MF differentiation. The specific phenotypes of fibroblasts infer their unique biological functions because their projection, spherical or flattened shapes can be influenced by surrounding microenvironments and intercellular interaction.^36^, ^41^ The ability of ECM degradation was enhanced in the coculture of MPCs and fibroblasts, which was also confirmed in the animal model of OSF. Since the interaction between MPCs and fibroblasts is dynamic, the therapeutic intervention that is instantly introduced may reverse the progress of fibrogenesis after tissue injury.

In the cell therapy based on transplantation of stem/progenitor cells, the donor age and gender are important factors to be optimized for clinical applications. In BM‐MSC, the high‐clonogenic samples were obtained from young female donors.^42^ Similarly, the studies of human MPCs demonstrated these cells could be harvested from donors of all ages and both gender, fast‐growing cells in the samples from young and female donors.^15^ In the current study, MPCs could be successfully isolated from human donors of different ages and genders. The most productive groups with greater cell‐yields were those harvested from the young donor, showing the compatible trend. These data describe the donor‐associated parameters pertinent to transplantation of MPC for cell therapy. The well‐defined condition and optimized protocols will give insight to feasible protocols for employing human MPCs for clinical applications.

A number of stem/progenitor cells originating from mesenchyme are competent for soft tissue repair and regeneration, and capable of regulating the process of fibrosis. MSCs are one of the progenitor cells that had been widely investigated.^43^ In addition to their potency of multi‐lineage differentiation, they can influence surrounding cells by paracrine mechanisms. The antifibrotic effect of BM‐MSC is principally mediated by the secreted molecules to stimulate associated signaling.^44^ ADSC secretes various bioactive factors to promote cell migration, proliferation, and differentiation. Their antifibrotic effects had been reported to depend on the secreted beneficial factors.^45^ Since both BM‐MSC and ADSC are potent in modulating muscle differentiation and fibrosis progression,^46^ they are compared parallelly with MPCs to clarify the similarity and divergence in genotyping. It was found that the molecular signatures of MPCs harvested by our methodology were similar. The cell populations of progenitors are usually assumed to be homogenous initially, nonetheless, they still possess remarkable heterogeneity.^47^ It is evident in many types of tissue‐specific stem cells including MPCs.^48^ The close relationship between BM‐MSC and MPC supports their potential in regulating the progression of fibrosis.

Cells utilized for tissue regeneration either directly contribute to the formation of new tissue or indirectly facilitate the progress through signaling regulation. In addition to being differentiated and directly engrafted into the damaged tissues, the progenitor cells also participate in tissue regeneration and remodeling through paracrine effects. The extracellular vesicles secreted by stem cells were reported to contain potent antifibrotic biologics for the treatment of fibrosis.^49^, ^50^ Secretome analyses reveal a broad range of the networks of protein–protein interaction involved in the process. During the crosstalk between fibroblasts and MPCs, increased secretome levels majorly by MPCs were proven to significantly change the behaviors of fibroblasts. Regarding VEGF, uPA, and MMPs that appear in the secretome, there is a close connection among them for ECM remodeling. VEGF directs pro‐uPA activation by interacting with VEGFR2, which depends on MMP2 activation.^51^, ^52^ The activated uPA subsequently transforms the pro‐enzyme plasminogen into plasmin that catalyzes the pro‐MMPs into activation forms, and plasmin itself can directly target ECM for degradation.^53^, ^54^ In a positive feedback loop, uPA promotes angiogenesis by upregulating the expression of VEGFR1 and VEGFR2.^55^ uPA and MMPs are implicated in ECM remodeling,^56^ and these effects disappear when uPA and MMP are inhibited.^57^ The mechanism of ECM remodeling of uPA relies on itself, or through generation of plasmin since activation of MMPs is required to be assisted by plasminogen.^58^ MMPs enhance VEGF production,^59^ and contribute to fibrosis resolution.^60^ When VEGF increases in the microenvironment, it effectively stimulates the production of metalloproteinases, tissue‐type and uPAs, and plasminogen activator inhibitor 1.^61^ When VEGF facilitates fenestrations in endothelial cells, it increases vascular permeability, monocyte chemotaxis, and macrophages function, which are beneficial for fibrosis resolution.^60^ The data presented here explain the potential mechanism accounting for the effect of reducing fibrosis by MPCs in other muscle injury models.^46^ Taken together, MPCs serve as a mediator of tissue remodeling, and the effects are prominent when the fibroblasts are present. The use of MPCs as treatment has a versatile and enormous beneficial impact on fibrosis resolution. When the therapeutic potential of VEGF, uPA and MMPs are considered, it is promising to target them in ECM remodeling to ameliorate fibrosis progression. In our secretome results, the third highest abundant soluble factor is IGFBP‐3. IGFBP‐3 and VEGF are closely implicated in angiogenesis, and IGFBP‐3 is beneficial to promote VEGF secretion.^62^ In addition, IGFBP‐3 is demonstrated to regulate fibrosis. The reciprocal interaction between bone morphogenetic proteins and IGFBP‐3 was observed in tissue remodeling by increasing MMP2.^63^ A reciprocal interaction between IGFBP‐3 and MMP was also found.^64^, ^65^ The role of IGFBP‐3 in VEGF and MMPs regulation is important in tissue remodeling, which may be one of the mechanisms accounting for fibrosis resolution mediated by MPC.

OSF is associated with inflammation followed by subsequent fibrosis of the deep layer of the tissue under oral submucosa to cause trismus.^66^ It is mediated by transformation of fibroblasts to become fibrotic‐associated MFs and promote production of ECM in the oral cavity. MFs are found in the early stage of OSF, and increases correspondingly with the progression of OSF.^17^ Accordingly, OSF is an appropriate model to test the capacity of MPCs by targeting MFs to ameliorate the degree of fibrosis in OSF. In OSF, the balance between MMPs and TIMPs is disturbed with increased ECM accumulation. TIMP‐1 and TIMP‐2 increase in the fibroblast isolated from OSF, whereas expression of MMP attenuates. Muscle is the abundant sources with normal cells adjacent to the pathological tissue of OSF. When MPCs were transplanted into the diseased oral submucosa, they survived in the in vivo microenvironment of OSF, differentiated toward muscle fibers, and successfully regulated MMPs for tissue remodeling and functional recovery. The data demonstrate the therapeutic potential of MPC for fibrosis. Further efforts will be required to optimize variables for clinical translation. According to our approach, it is feasible to harvest sufficient MPCs from the donor with different demographic background, which overcome the major limitation of cell availability in clinical applications.^46^

Many studies have demonstrated the clinical potential of myogenic stem/progenitor cells for treating impaired muscle tissues.^67^, ^68^ Despite their therapeutic potential, the effect of tissue integration of these transplanted cells remains elusive. The studies of stem cell transplantation for the treatment of myocardial infarction has confirmed the importance of muscle integration of transplanted cells.^69^, ^70^ The heterogeneity of donor cells and inappropriate cell fusion and adhesion to local host tissue may lead to adverse tissue function.^71^ Although the overall survival of transplanted cells remained low, and the integration of transplanted cells remained controversial, functional improvement was observed in many diseases after transplantation of muscular progenitor cells.^72^, ^73^ It is proposed that milieu‐dependent effects may be more important than the direct differentiation and integration of the transplanted progenitor cells.^74^ In the current study, the significant physiological recovery and muscle regeneration were found in vivo may warrant the effect of MPC treatment. Whether the effect mediated by MPCs is derived from the direct differentiation and integration with local muscular tissue, or from the environmental stimuli on the host environment may need further study to validate.

## 4 MATERIALS AND METHODS

### 4.1 Cell preparation and culture

For isolation and primary culture of hMPCs and fibroblasts (hPFs), the normal soft tissue was harvested from the redundant parts of the surgical specimens after the operation of benign diseases with the approval of Institutional review board. The procedures of tissue processing to isolate hMPCs and hPFs followed the standard protocols previously published.^9^, ^12^, ^13^, ^15^, ^75^ Briefly, the tissue was first minced and digested by type I collagenase 0.2% (w/v) (Sigma‐Aldrich) and dispase 0.4% (w/v) (Corning) for 120 min at 37°C. After digestion, repetitive rigorous pipetting was used to release muscle fibers and fibroblasts. The tissue pellets were prepared by filtration and centrifugation, and then suspended with medium on a type I collagen coated dishes (BD Biosciences Clontech). After 24 h, the supernatant containing nonadhered MPCs was transferred to another new dish, and the original dish was used for hPFs collection. The procedures were repeated every 2 days to increase the purity of MPCs and hPFs populations. The growth medium and differentiation medium were prepared as the same as those previously described.^9^, ^12^, ^13^, ^15^ To prepare MFs without the interference caused by cytokine supplement in culture, the cell‐density plating method was employed.^21^, ^22^, ^23^, ^24^ During coculture of hPFs or MFs with MPCs, cells were seeded at 1 × 10^5^ on the coverslips. When cells attached, transferred the coverslips to six well plates and 40 μm cell strainers. The different cells were placed separately in two compartments of the transwell system. The culture lasts for 16–24 h, then cells were subjected to subsequent analyses.

### 4.2 Array and data analyses

The cells were harvested from the coculture and controls. Total RNA was extracted by the RNeasy Mini kit (Qiagen, Valencia, CA). The yield and quality control of mRNA samples were examined before microarray analysis. The Human Whole Genome OneArray (Phalanx Biotech, HsinChu, Taiwan) was used. Three repeats from independent specimens were enrolled. The raw data were confirmed with the Pearson correlation coefficient for technical reproducibility. Differentially expressed genes were identified when the expression was in the spot with the following criteria including a log_2_ ratio ≥1 or a log_2_ ratio ≤−1 and a *p*‐value <0.05.^76^ To correlate the predefined gene sets with phenotypes, data were analyzed and ranked by GSEA software from the Broad Institute with default setting.^77^ The gene sets ranked by ratio were subjected to KEGG database and leading‐edge analysis for uncovering genes that contribute to the enrichment scores. To validate the similarity between MPC and other mesenchymal progenitor cells, we retrieved microarray data of BM‐MSC and ADSC from NCBI GEO database, and these data were further analyzed by the Multi Experiment Viewer microarray analysis software.

### 4.3 Immunofluorescence

Cells were cultured on coverslips and were fixed with 4% paraformaldehyde for 20–30 min, permeabilized by 0.4% Triton X‐100, and blocked in 1% BSA/1x PBST at room temperature for 1 h. Primary and secondary antibodies were diluted in a ratio of 1:1000 and 1:10,000 with blocking buffer, respectively. The information of primary antibodies used in current study was listed as following: vimentin (ab24525, Abcam), α‐SMA (A2547, Sigma), collagen I (C2456, Sigma), FN1 (ED‐A) (ab6328, Abcam), MMP1 (ab137332, Abcam), MMP2 (GTX104577, GeneTex), MMP8 (GTX61732, GeneTex), MMP9 (ab38898, Abcam), and MMP13 (ab39012, Abcam). For secondary antibodies: anti‐rabbit Dylight 488 (SA5‐10038, Thermo Fisher Scientific, Waltham, MA), anti‐chicken Dylight 550 (SA5‐10071, Thermo Fisher Scientific), anti‐mouse Dylight 633 (35512, Thermo Fisher Scientific), and mouse Dylight 488 (35503, Thermo Fisher Scientific). Samples were immunostained with primary antibody (1:200) at 4°C overnight, and further incubated with secondary antibodies (1:200) conjugated with Dylight at room temperature for 1 h. Nuclei were counterstained with DAPI (1:1000), and the samples were mounted with anti‐fade solution. Fluorescent images were obtained using Leica TCS SP8 confocal microscope and analyzed by ImageJ software to determine the fluorescent intensity.^78^, ^79^, ^80^

### 4.4 Semi‐quantitative and quantitative real‐time PCR

Total RNA was extracted from tissues or cells using RNeasy mini kit (Qiagen) as manufacturer's instruction, and reverse transcribed to complementary DNA (cDNA) with RevertAid First Strand cDNA Synthesis Kit (Thermo Fisher Scientific). The qRT‐PCR was performed by iQ SYBR green supermix (Bio‐Rad) and was run in triplicate using gene‐specific primer pairs for β‐actin as endogenous controls. Results were analyzed by Applied Biosystems (ABI) Real‐Time PCR System, and the data were normalized against housekeeping gene β‐actin and analyzed with the comparative threshold cycle method. For semiquantitative RT‐PCR, PCR reactions for cDNA amplification followed our standard protocols.^76^, ^81^ The PCR products were subjected to gel electrophoresis for confirming the size of desired products. The final results were obtained by triplicate reactions and three‐time repeats.

### 4.5 Zymography

To measure the ECM proteolytic activity in the secretome harvested from the coculture of MPC and fibroblasts, zymography was applied to detect the function of MMPs. In‐gel zymography was employed using the media retrieved from fibroblasts cultured with or without MPCs. The medium was centrifuged to remove cell debris, lyophilized, and resuspended in water. Equal levels of protein were determined before being loaded to each lane. MMP activity was determined by in‐gel zymography using gelatin or collagen as the components to prepare the gel. After separation, gels were washed, renatured with Triton X‐100, and incubated with protease inhibitors. The proteolytic bands were visualized by Coomassie brilliant blue and the densitometry was inverted for black and white to make it clear. The area digested in the gel represented the proteolytic activity of indicated MMPs with corresponding molecular weight. The regions were further quantitated by ImageJ for comparative analyses.

To directly identify the proteolytic activities of fibroblasts or MFs when culture with or without MPC, in situ zymography was applied using dye‐quenched (DQ) labeled substrates (Thermo Fisher Scientific). The experiments were conducted following the protocol provided by the manufacture. The cells (1 × 10^4^) were seeded on the DQ‐substrate (20 μg/ml) coating slides for coculture. After culture, the slides were fixed and counterstained with DAPI for the nucleus. Fluorescence of proteolytic activity was imaged, quantified by ImageJ program, normalized and corrected by subtraction to the negative control, and quantitatively compared.^82^

### 4.6 Proteomic analyses

The supernatants were collected from the fibroblasts cultured with or without MPCs for 48 h, and were subjected to proteomic analyses using the Proteome Profiler Human Array (R&D, Cat. # ARY007) according to the manufacture's instruction. In brief, these samples were loaded onto the membrane spotted with the antibodies for specific markers. After overnight incubation, the results for different substrates were developed and detected. Quantification of the pixel densities was determined using AlphaEaseFC 4.0 software. Each spot was analyzed by Vision Works LS software (UVP, CA). The data were first normalized to positive control, and presented as the fold changes.

### 4.7 MPCs treatment in the OSF animals

The animal model of OSF was established to evaluate the effect of MPCs in vivo. In the current model, MPCs were harvested from human samples and transplanted into the murine model as xenogeneic grafts. The chemical method reported to successfully set up OSF animal model was followed and modified using adult CB17ICR‐SCID mice, with the approval of by the Animal Care and Use Committee of the institute.^39^ Then, 4% phenol solution was injected into the submucosa of both sides of oral cavity by 30G‐needles. One month later, MPCs were prepared (1 × 10^7^ cells/ml) and injected into the treated sites. Sham operations followed the same procedures without injection of MPCs in the OSF group. The physiological parameters of measuring the mouth opening width were recorded and compared with the control (OSF) and wide‐type groups after 90 days of MPC treatment. The mouth opening was measured based on the length between the roots of the upper and lower incisors. All animals were sacrificed for tissue harvest. For histological analyses of harvested tissue, sampling of the fields for examination followed the published methodology.^83^ More than 10 fields under the pathological examinations were randomly selected from each side, and at least three sections of each specimen were evaluated. Submucosal collagen accumulation was demonstrated using Masson's trichrome staining. The fibrosis scoring system that had been widely accepted for evaluating the subepithelial fibrosis was applied.^84^, ^85^ Briefly, the degree of fibrosis was scored by the histological features of collagen and associated cell density. The scores were evaluated among these groups to provide the quantitation of the fibrosis that is principally composed of collagen fibers. The existence of the muscle fiber differentiated from injected human MPCs was confirmed by RT‐PCR and immunofluorescent staining using the markers derived from human origins. Expression of the MMPs in the harvested tissue was assessed by immunofluorescent staining, and the proportion of positive‐stained fibroblast was quantitatively compared between the treated (MPC + OSF) and the control (OSF) groups.

### 4.8 Statistics

All measurements were averaged from more than three independent experiments for indicated experimental settings. The continuous data were presented as the mean and standard deviation that were compared by *t* test. The categorical data were analyzed using chi‐square or Fisher exact tests. In this study, the statistical analysis was calculated with GraphPad prism ver.9.3.0, and the results of charts and statistics were further confirmed by Excel and other statistic software. The statistical significance labeled when the *p*‐value was less than the indicated values: *<0.05; **<0.01; ***<0.001; ****<0.0001. The statistical analysis was based on GraphPad Prism (San Diego, CA).

### 4.9 Illustration

The illustration demonstrated in the main and supplementary figures were created with Biorender.com.

## 5 CONCLUSION

The current study confirms the novel properties of MPCs to reverse the fibrosis progression by regulating the transformation and activities of fibroblasts. In addition to being differentiated into mature muscle fibers, MPCs are competent to influence fibroblasts and remodel the fibrosis processes simultaneously after tissue injury. The data revealed the mechanism of MPC in mediating fibrosis regression through paracrine effects by regulating the activities and crosstalk of the associated molecules including MMPs, uPA, and VEGF, which promoted ECM degradation and tissue modeling. The augmented effect was further confirmed in vivo when MPCs were applied in the OSF animal models. Since MPCs could be successfully harvested from all human samples regardless of demographic background, they are demonstrated to be the promising candidates for development of the cell therapy for antifibrosis and tissue regeneration.

## AUTHOR CONTRIBUTIONS

**Ya‐Chuan Hsiao:** Conceptualization (lead); data curation (equal); formal analysis (equal); funding acquisition (supporting); investigation (lead); methodology (equal); project administration (equal); resources (equal); software (supporting); validation (equal); visualization (equal); writing – original draft (equal); writing – review and editing (equal). **I‐Han Wang:** Data curation (supporting); formal analysis (supporting); investigation (supporting); methodology (supporting); project administration (supporting); software (supporting); validation (supporting); visualization (supporting); writing – original draft (supporting); writing – review and editing (supporting). **TSUNG‐LIN YANG:** Conceptualization (lead); data curation (lead); formal analysis (lead); funding acquisition (lead); investigation (lead); methodology (lead); project administration (lead); resources (lead); software (lead); supervision (lead); validation (lead); visualization (lead); writing – original draft (lead); writing – review and editing (lead).

## CONFLICT OF INTEREST

The authors declare that there is no conflict of interest that could be perceived as prejudicing the impartiality of the research reported.

### PEER REVIEW

The peer review history for this article is available at https://publons.com/publon/10.1002/btm2.10439.

## Supporting information


**APPENDIX S1** Supporting Information

## Data Availability

The authors approve that the data supporting the findings are available in the main article and supplementary information.
