# Supplementary material for: Fibrotic remodeling and tissue regeneration mechanisms define the therapeutic potential of human muscular progenitors
Source: Bioeng Transl Med. 2022 Nov 26;8(2):e10439. doi: 10.1002/btm2.10439 (PMC10013817; doi:10.1002/btm2.10439)
Supplement: Supplementary file 1 — APPENDIX S1 Supporting Information [file BTM2-8-e10439-s001.docx]

**Materials and Methods**

**Cell proliferation**

To analyze hPF and MF cell proliferation, Ki67 staining was conducted. Co-cultured or non-cocultured cells on coverslips were fixed in 4% PFA for 30 min, the coverslips were wahsed with PBS for 4×10 min. 1% BSA / 1×PBST (0.4% Triton X-100) was used as blocking reagent at RT for 1h. Then 1:200 of anti-rabbit monoclonal Ki67 (ab16667, Abcam) antibody was used and incubated overnight. Next day, coverslips were washed with PBST for several times and incubated with 1:200 of anti-rabbit Dylight 488 (SA5-10038, Thermo Fisher Scientific) and DAPI (1:1000) for 1h at room temperature. The coverslips were mounted with anti-fade medium and took pictures by fluorescence microscope. The data were analyzed for comparison.

**VEGF and TGF-β reagents and assays**

Human VEGF neutralizing antibody (MAB293-100), recombinant human VEGF 165 protein (293-VE-010/CF) and recombinant human TGF-β protein (240-B-010/CF) were purchased from R&D Systems. For VEGF-related experiments, the VEGF neutralizing antibody (10 ng/mL) or recombinant human VEGF 165 protein (10 ng/mL) were added in the single culture of hPF/MF or the co-cultures of hPF/MF with MPC separately. The control and experimental groups were incubated with the VEGF recombinant protein, or VEGF neutralizing antibody for for 8 hours. For TGF-β-induced MF assay, the recombinant human TGF-β protein was applied in a concentration of 2 ng/mL for 1-day treatment. The cells were further subjected to subsequent analyses.

**Flow cytometry**

To confirm the impact of MPC on the expression of fibrosis-associated markers, cultured hPFs and MFs with or without MPCs were collected with trypsin-EDTA. Cells were centrifugated and resuspended. The cells were divided into 4 groups for control, and the following antibodies with predetermined optimal amounts were applied as follows: α-SMA/FITC, desmin/FITC, and vimentin/FITC. After incubation, the samples were collected, washed, and subjected to FACScan immediately. Cells of each group were analyzed by CellQuest pro software (BD Biosciences).

**Figure legends**

**Fig. S1: The global gene expression profiles of MPC, ADSC, and BM-MSC.** Gene Ontology (GO) term enrichment analysis of differential expressed genes processes with **(a)** KEGG pathway database (a, left panel: MAPK pathway; right panel: focal adhesion pathway), and **(b)** gene symbol database (b, left panel: cell proliferation; right panel: cell cycle).

(ADSC: adipose derived stem cells; BM-MSC: bone marrow mesenchymal stem cells)

**Fig. S2. Induction and characterization of myofibroblasts during *in vitro* culture**

**(a)** Representative immunofluorescent imaging of myofibroblasts differentiation using cell density method on day 1, day 6 and day 6 of the third passage (d6-P3), with different cellular densities **(b)** Quantitative analyses of desmin, α-SMA and vimentin expression of cultured cells. **(c)** Induction of myofibroblast differentiation by TGF-β treatment. **(d)** Quantitative analyses of desmin, α-SMA and vimentin expression of the cultured cells. (Scale bar: 100 μm; n≥3 biological replicates)

**Fig. S3. Expression of fibrosis-associated markers with or without MPC coculture in fibroblasts and myofibroblasts.**

Flow cytometry analyses of **(a, d)** α-SMA, **(b, e)** desmin and **(c, f)** vimentin in hPF or MF respectively, with or without MPC culture. Quantitative data were presented as the mean fluorescence intensity (MFI) indexes. (n≥3 biological replicates)

**Fig. S4. The phenotypes of MPCs were preserved without differentiation toward MF during coculture.**

**(a)** The typical markers of muscle precursor including Myf5, MyoD, Myogenin and Pax7 were immunofluorescently stained in MPC without (left panel) or with MPC co-culture (right panel). Scale bar:50 μm. **(b)** Single-culture and co-culture of hPF, MF and MPC were stained with desmin, α-SMA and vimentin. α-SMA and vimentin showed positive expression in MF. the vimentin was all positive in MPC, and the single-culture of MPCs showed strong staining patterns compared with stains in the co-culture. Scale bar:200 μm. **(c)** The relative expression levels of vimentin in the MPC, MPC co-cultured with hPF, and MPC co-cultured with MF were compared. The analyzed cells for comparison were underlined. (n≥3 biological replicates)

**Fig. S5. Real-time PCR for TGFβ and TIMP families in the coculture of fibroblasts/myofibroblasts and MPC.**

The fold changes of the gene expression of **(a)** TGFβ1 and TGF-β receptors, and **(b)** TIMP1 and TIMP2 in the hPF culture with MPC. The fold changes of the gene expression of **(c)** TGFβ1 and TGFβ receptors, and **(d)** TIMP1 in the MF culture with MPC. (n≥3 biological replicates)

**Fig. S6. Immunofluorescence staining and quantification of Ki67 co-expressed cells in hPFs or MFs culture with or without MPC.**

**(a)** hPF or **(b)** MF cultured with or without MPCs were stained by Ki67 and DAPI for proliferation assays. In the images, the white arrowheads indicate the Ki67-positive cells, whereas the arrows label Ki67-negative cells. Scale bar: 100 μm. (N.S.: non-significance; n≥3 biological replicates)

**Fig. S7.** **The heat map analyses of the protein levels after MPC co-culture with hPF**. A full list of heat map analyses visualizes the fold-changes of protein levels after MPC co-culture with hPF. Measurements of co-cultures were carried out four times and normalized to the control group of hPF alone. Colors are designated as fold-increase changes, ranging from the highest levels (red) to the lowest levels (blue).

**Fig. S8. Regulation of DQ-gelatin degradation of MMP9 expression by VEGF in hPF/MF with MPC.**

**(a)** Degradation of DQ-gelatin (green fluorescence) in the single culture of hPF/MF and the co-cultures of hPF/MF with MPC treated by VEGF recombinant protein or VEGF neutralizing antibody. Quantification of fluorescent intensity of **(b)** hPF and **(c)** MF groups. **(d)** IF imaging of MMP9 in in the single culture of hPF/MF and the co-cultures of hPF/MF with MPC treated by VEGF recombinant protein and VEGF neutralizing antibody. Quantification of fluorescent intensity of **(e)** hPF and **(f)** MF groups. (Scale bar: 50 μm; n>3 biological replicates)

**Fig. S9. Schematic illustration of the proposed effect of MPC on fibrosis.** MPCs secret essential cues including MMPs and VEGF-associated factors to regulate the behaviors of hPFs and MFs, which is beneficial for fibrosis suppression.

(MPC: muscle precursor cells; MF: myofibroblasts; hPF: human primarily cultured fibroblasts; MMPs: Matrix metalloproteinase; VEGF: Vascular endothelial growth factor)

**Literature Cited**

1. Pattyn, F., P. Robbrecht, et al. RTPrimerDB: the real-time PCR primer and probe database, major update 2006. *Nucleic Acids Res.* 2006;34:D684-688.

| 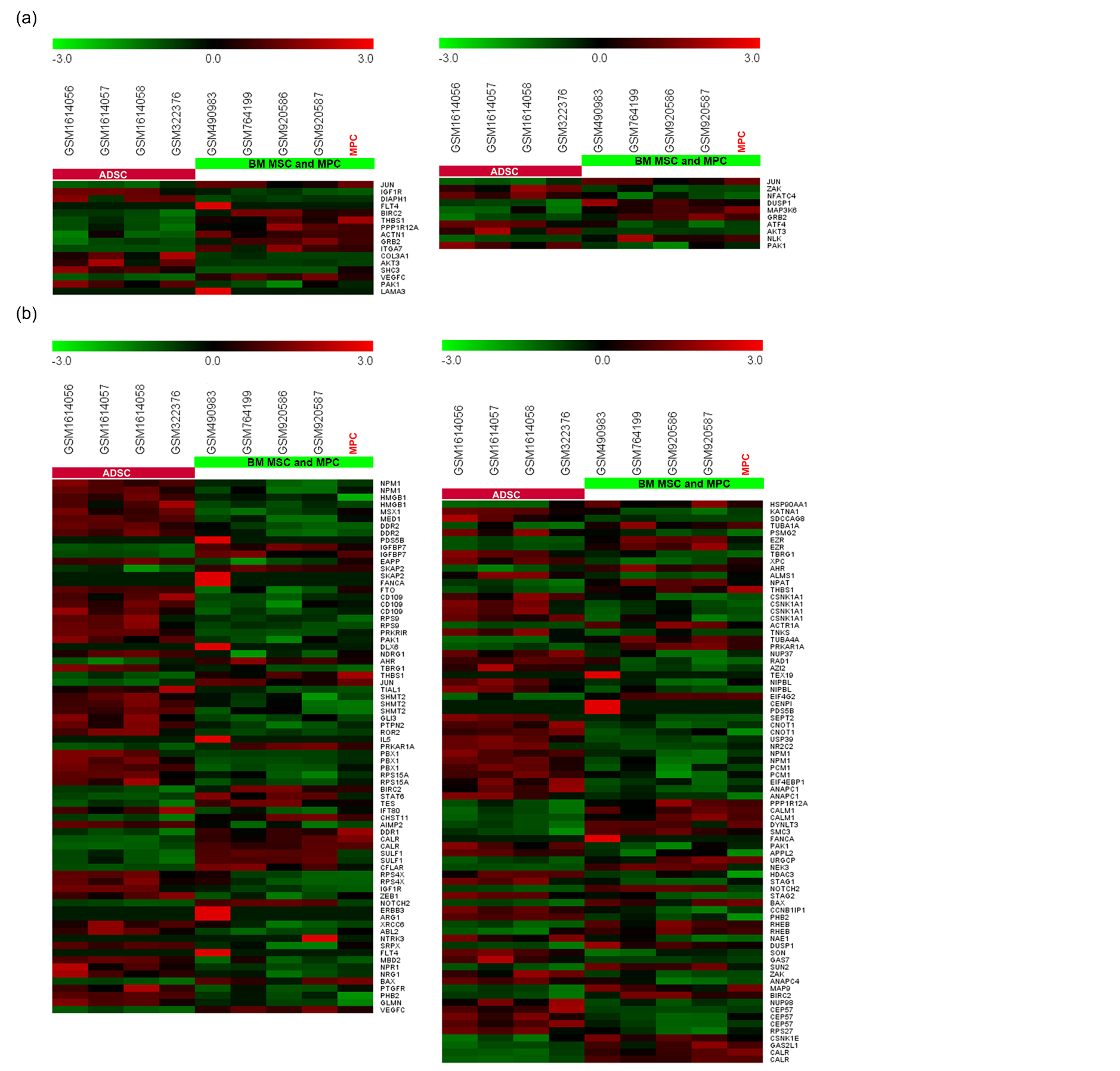 |
| --- |
| **Fig. S1: The global gene expression profiles of MPC, ADSC, and BM-MSC.** |
| 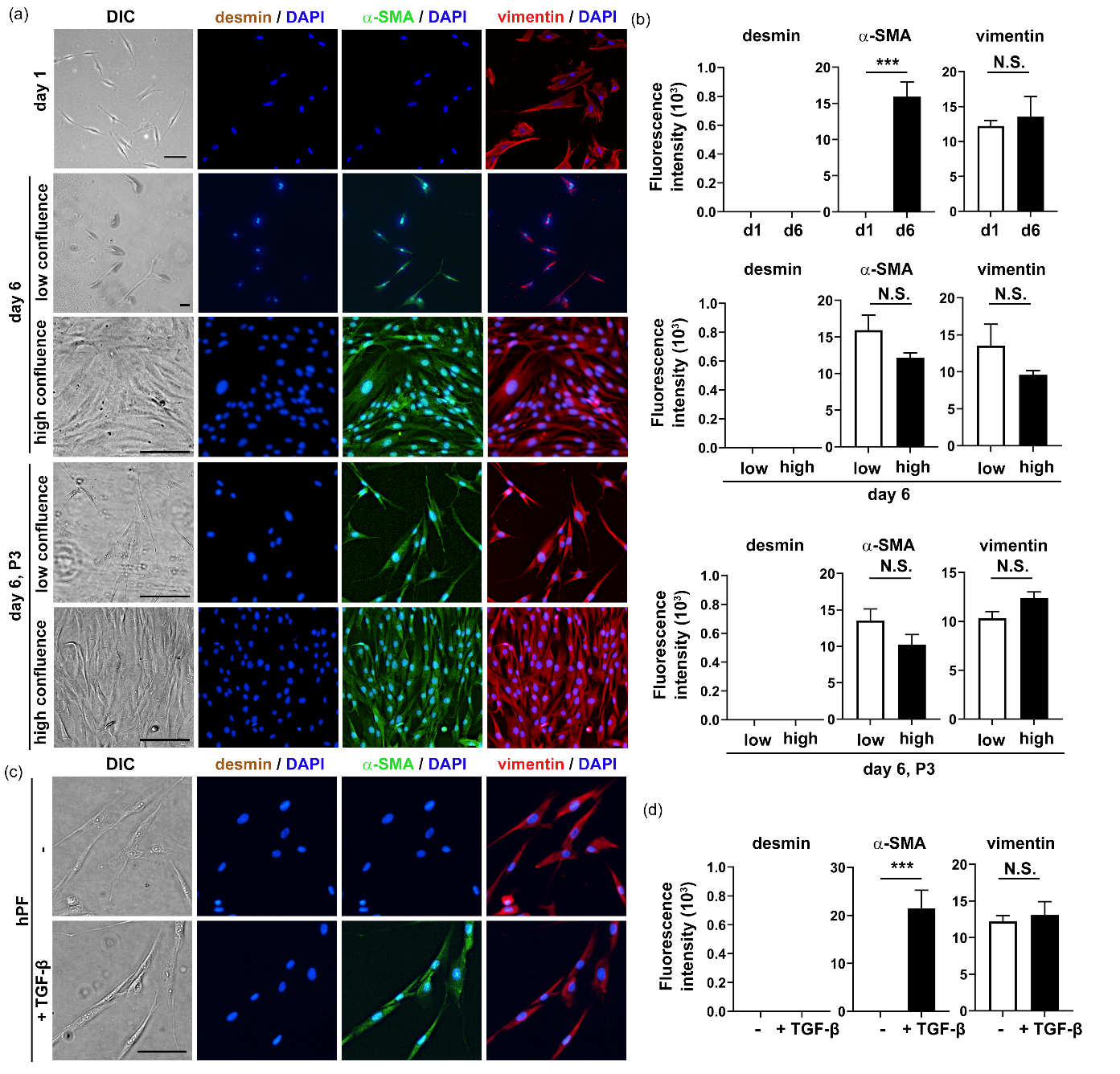 |
| **Fig. S2. Induction and characterization of myofibroblasts during *in vitro* culture** |
| 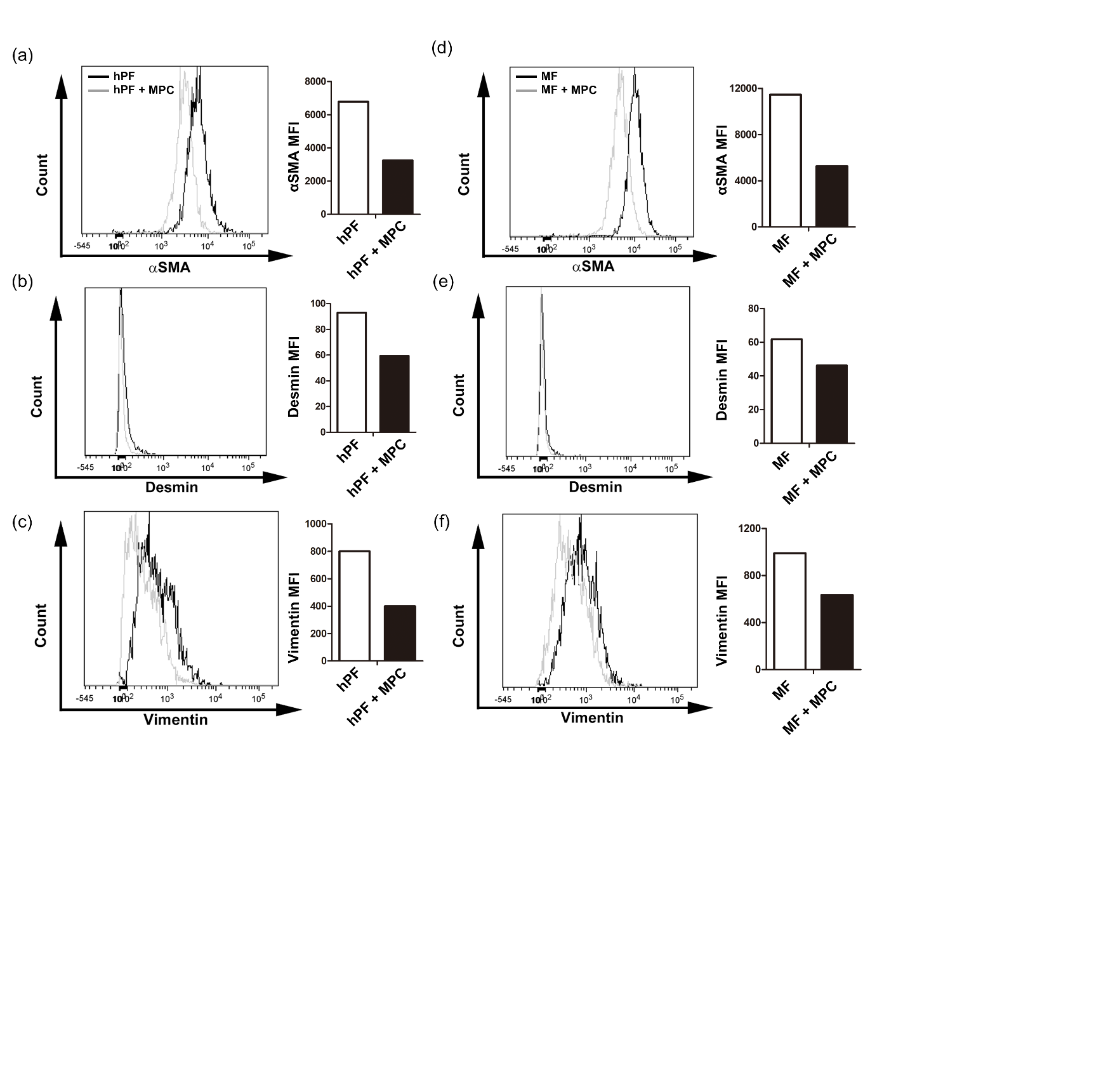 |
| **Fig. S3. Expression of fibrosis-associated markers with or without MPC coculture in fibroblasts and myofibroblasts.** |

| 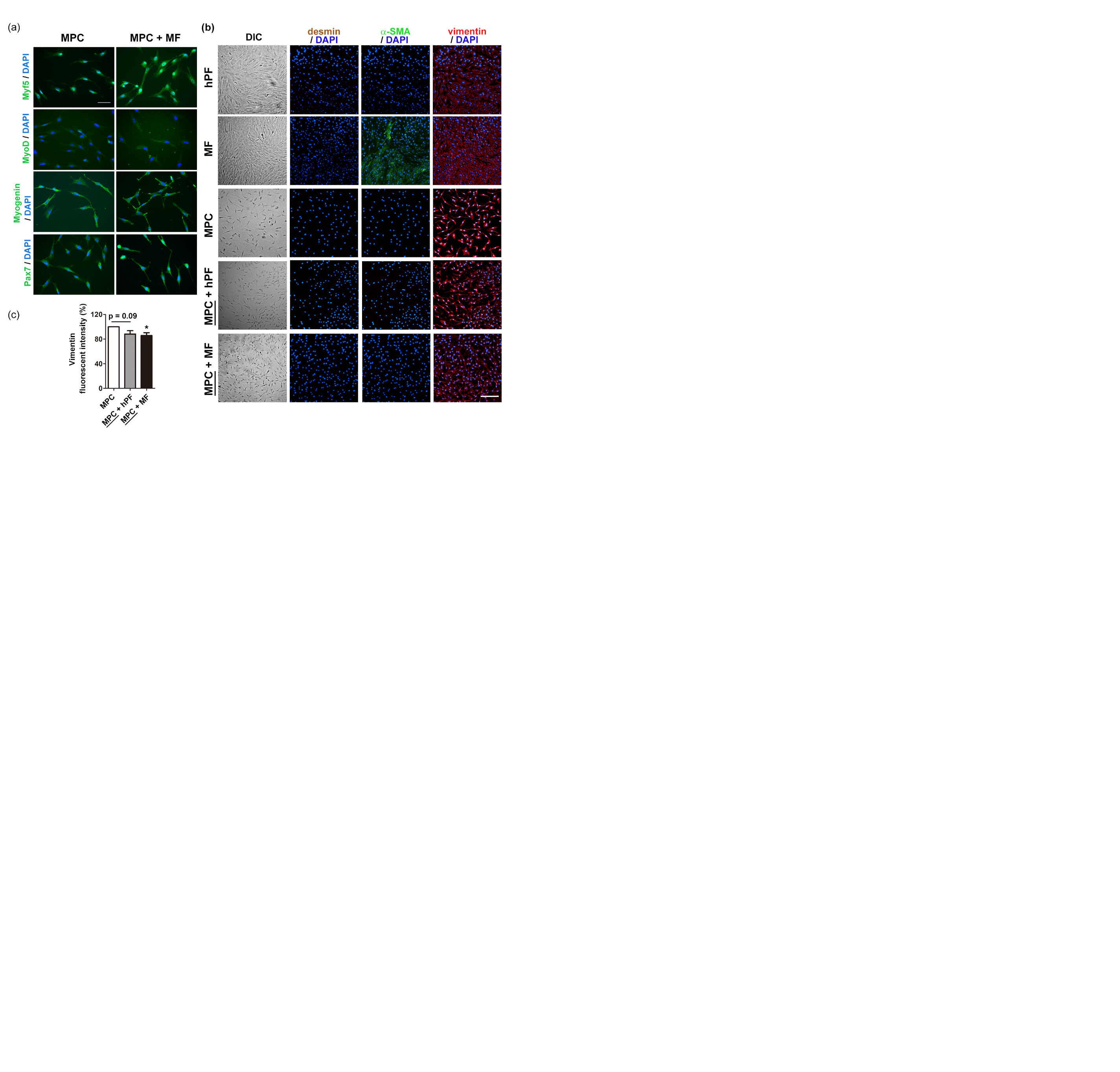 |
| --- |
| **Fig. S4. The phenotypes of MPCs were preserved without differentiation toward MF during coculture.** |
|  |

|  |
| --- |
| 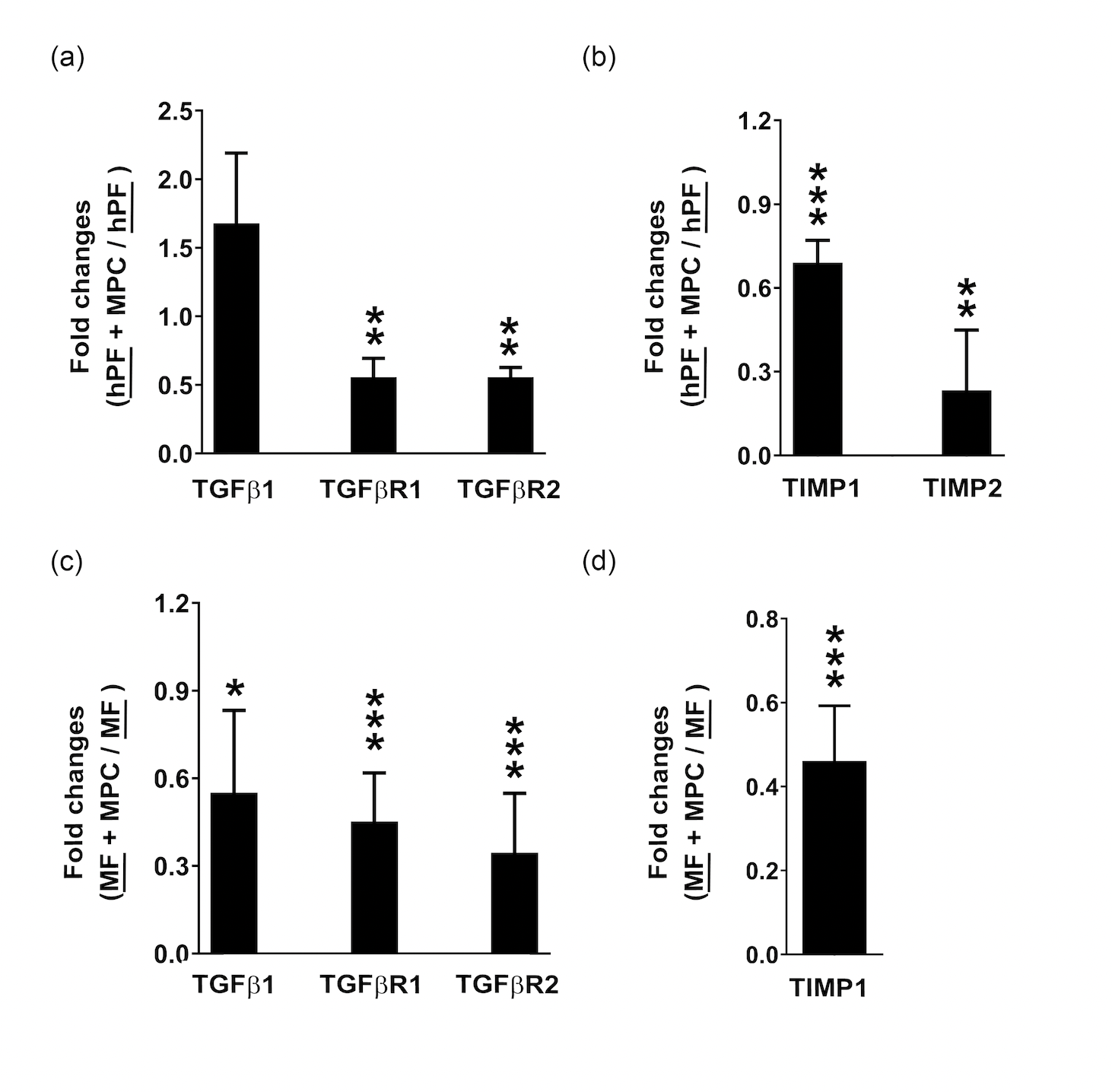  **Fig. S5. Real-time PCR for TGFβ and TIMP families in the coculture of fibroblasts/myofibroblasts and MPC.** |

| 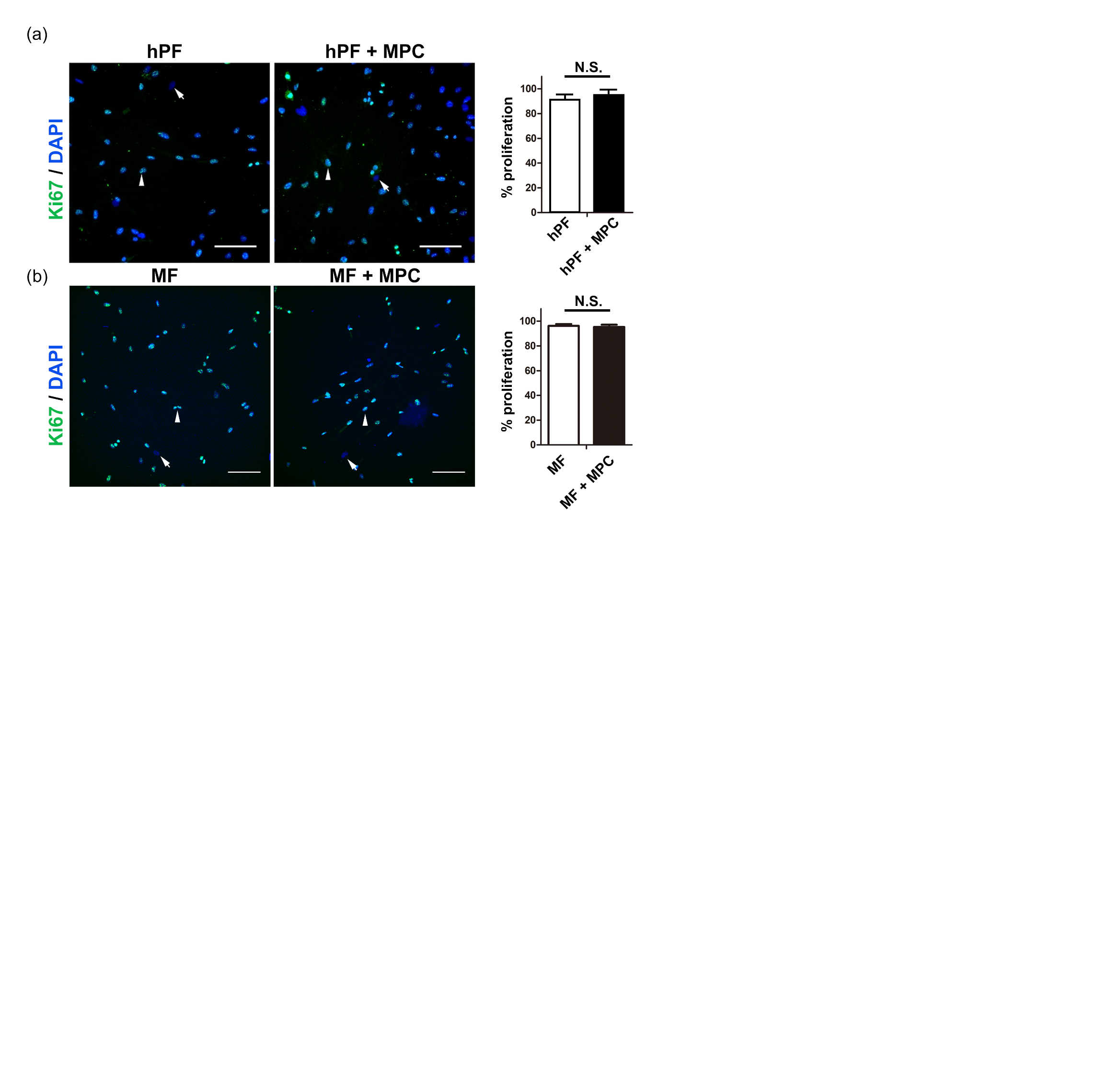  **Fig. S6. Immunofluorescence staining and quantification of Ki67 co-expressed cells in hPFs or MFs culture with or without MPC.** |
| --- |

| 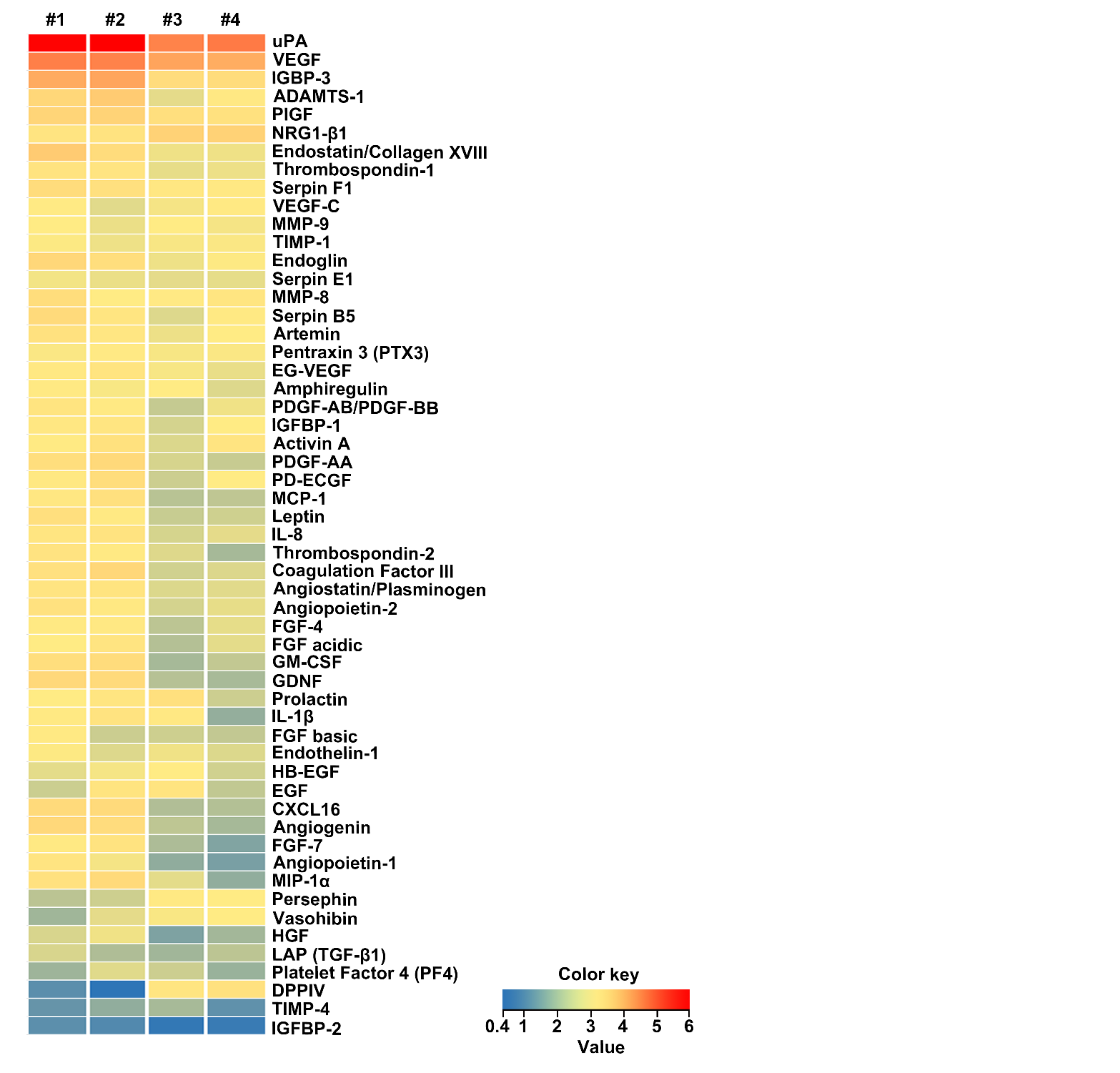 |
| --- |
| **Fig. S7. The heat map analyses of the protein levels after MPC co-culture with hPF.** |

**
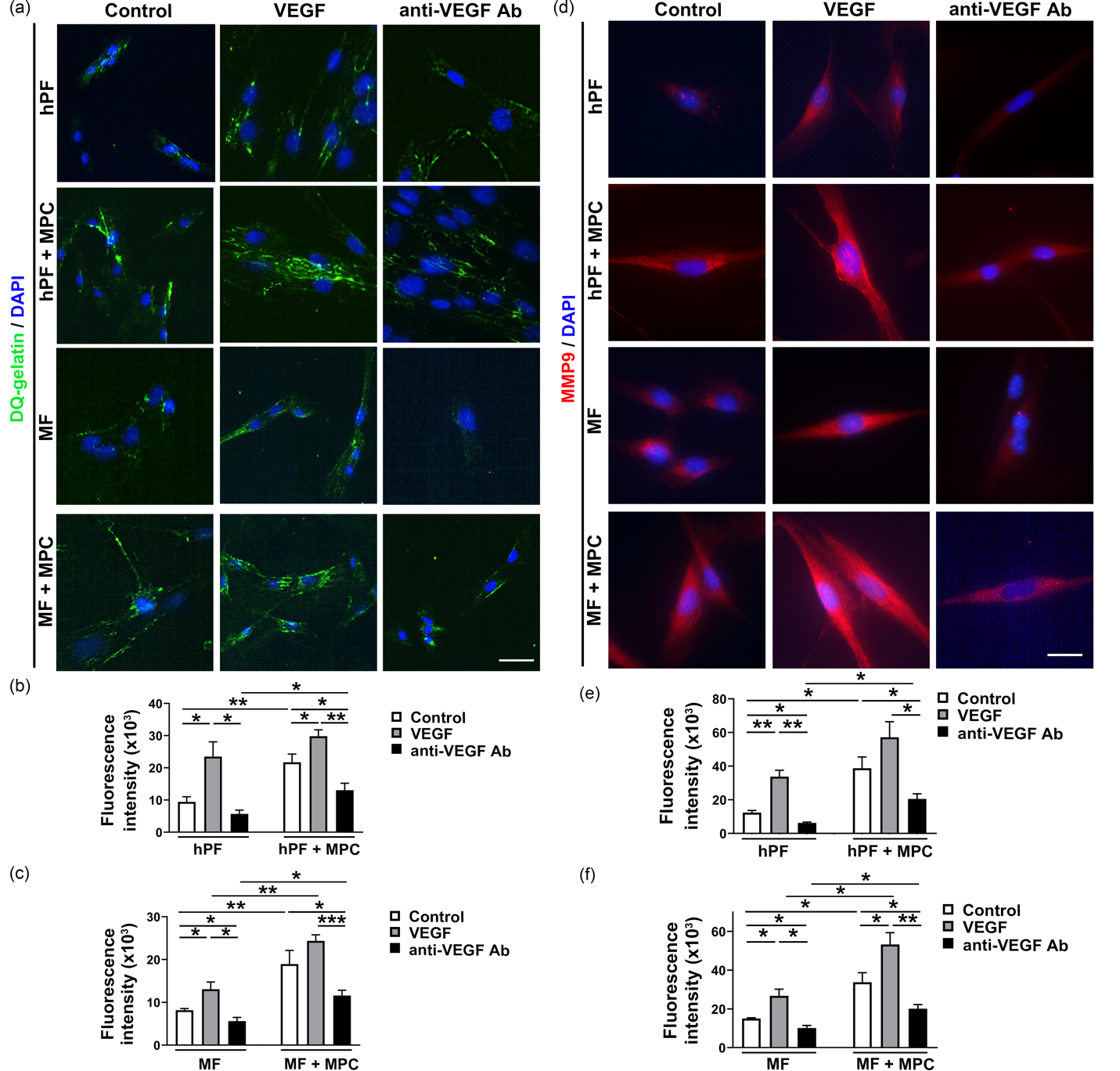
**

**Fig. S8. Regulation of DQ-gelatin degradation of MMP9 expression by VEGF in hPF/MF with MPC**

| **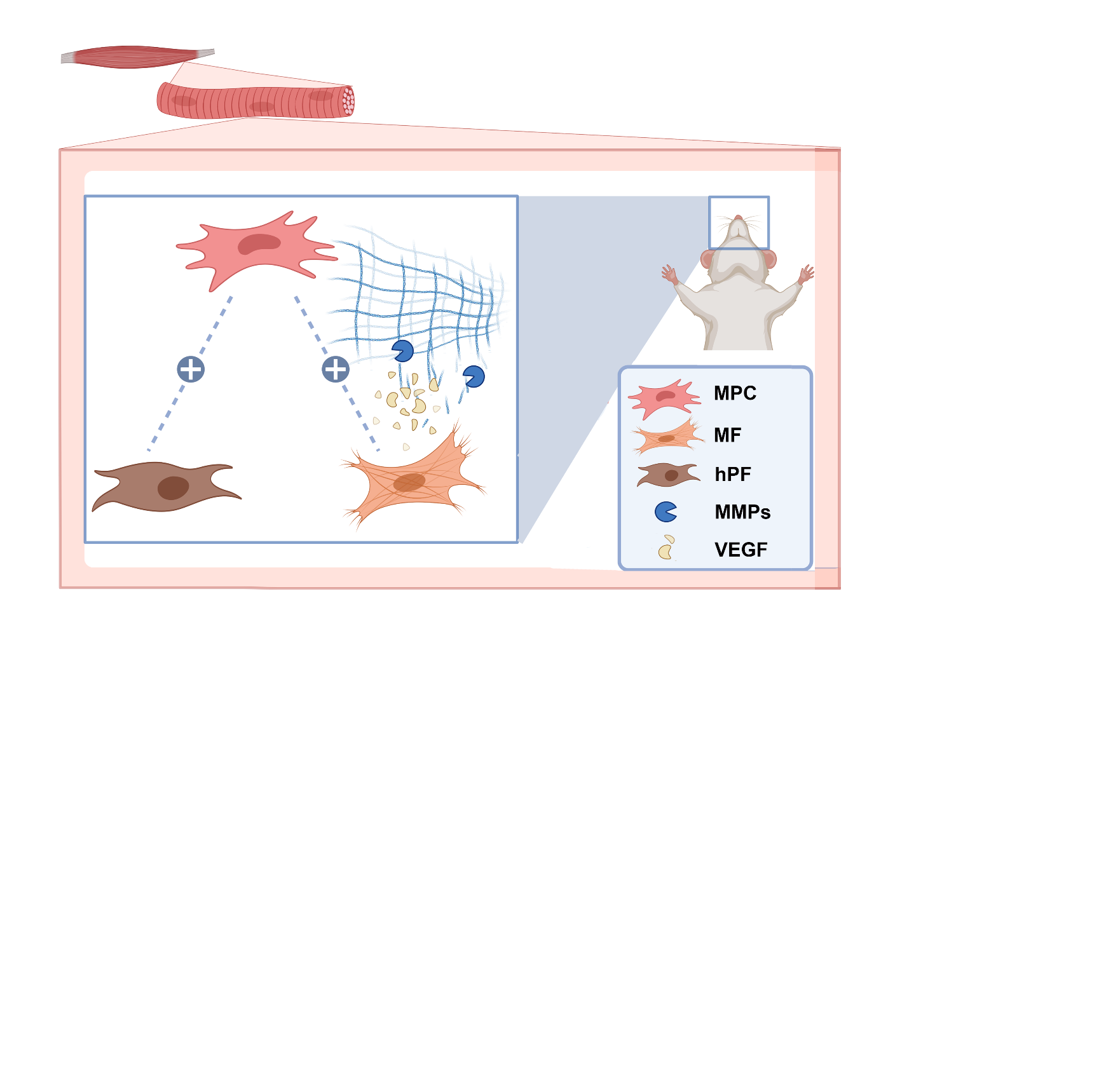** |
| --- |
| **Fig. S9. Schematic illustration of the proposed effect of MPC on fibrosis.** |

| **Table S1. List of real-time PCR primers**   \|  \| Forward primer (5' -> 3') Reverse primer (5' -> 3') \| Amplicon size (bp) \| \| Ref Seq \| \| \| --- \| --- \| --- \| --- \| --- \| --- \| \| MMP1 \| GAGAGCAGCTTCAGTGACA \| 183 \| \| NM_001145938.1 \| \| \|  \| ACAAGGTTGACTTTATTCCAAACA \|  \| \|  \| \| \| MMP2 \| TGCGACCACAGCCAACTACGAT \| 159 \| \| NM_001302510.1 \| \| \|  \| CTTGGTGTAGGTGTAAATGGGTGCC \|  \| \|  \| \| \| MMP8 \| GAAGACAGAGGCAGAGATTC \| 121 \| \| XM_011542836.2 \| \| \|  \| CACACTTGAAGGCTATTGGA \|  \| \|  \| \| \| MMP9 \| GTGAAGGCGCAGATGGTGGA \| 130 \| \| NM_004994.2 \| \| \|  \| AGTAGAAGCGGTCCTGGCAGAAA \|  \| \|  \| \| \| MMP10 \| GAAGAAGATGAGCCTTGC \| 127 \| \| NM_002425.2 \| \| \|  \| AATACATTCTCTCACCTATTGC \|  \| \|  \| \| \| MMP11 \| CCTGCATCTGTCTGCCTTCT \| 197 \| \| ^1^ \| \| \|  \| GCTTTGGAGGATAGCAGTGC \|  \| \|  \| \| \| MMP13 \| AAGCAGTCTACTAGATTGTGAT \| 180 \| \| NM_002427 \| \| \|  \| CAACAGTGTCTCTGAGCA \|  \| \|  \| \| \| MMP14 \| CTGCCGAGCCTTGGACTGTCA \| 174 \| \| NM_004995.3 \| \| \|  \| CGGTCATCATCGGGCAGCAC \|  \| \|  \| \| \| MMP16 \| TGGCAGCACAAGCACATCACTT \| 84 \| \| NM_005941.4 \| \| \|  \| AGGCACGGCGAATAGCTTTACG \|  \| \|  \| \| \| MMP26 \| TTGACAAGAATGAACACTG \| 142 \| \| NM_021801 \| \| \|  \| TCGTGATACCAGTAAGTG \|  \| \|  \| \| \| TIMP1 \| CAACCAGACCACCTTATACCAGCGT \| 146 \| \| NM_003254.2 \| \| \|  \| CGGTTGTGGGACCTGTGGAAGTATC \|  \| \|  \| \| \| TIMP2 \| AAGGAAGTGGACTCTGGAAACGACA \| 164 \| \| NM_003255.4 \| \| \|  \| CTTCTTTCCTCCAACGTCCAGCG \|  \| \|  \| \| \| Col4a1 \| AAGGCGATCAAGGCGTCCC \| 140 \| \| XM_011521048.2 \| \| \|  \| TGGCAGTCCCTGAAGCCCTT \|  \| \|  \| \| \| Col1a1 \| TGCTCCTGGCAAAGATGGACTCA \| 194 \| \| XM_005257058.4 \| \| \|  \| CACCATCGTGAGCCTTCTCTTGAG \| \|  \| \|  \| \| TGFb1 \| CCGCGTGCTAATGGTGGAAA \| \| 158 \| \| XM_011527242.2 \| \|  \| ACTTGAGCCTCAGCAGACGCA \| \|  \| \|  \| \| TGFbR1 \| TGCTGACATCTATGCAATGGGCTT \| \| 181 \| \| XM_024447658.1 \| \|  \| CCATCTGTTTGGGATATTTGGCCT \| \|  \| \|  \| \| TGFbR2 \| TGGTGCTCTGGGAAATGACATCTC \| \| 120 \| \| XM_011534045.3 \| \|  \| ACACGTTGTCCTTCATGCTTTCGA \| \|  \| \|  \| \| ACTA2 \| ACGTTTCCGCTGCCCAGAGA \| \| 176 \| \| NM_001613.3 \| \|  \| GGTCGGCAATGCCAGGGTAC \| \|  \| \|  \| \| FN1 \| GGTGCCATGACAATGGTGTGAAC \| \| 180 \| \| XM_024452771.1 \| \|  \| TCTGCCACTGTTCTCCTACGTGGTA \| \|  \| \|  \| \| VEGFa \| CTACCTCCACCATGCCAAGT \| \| 109 \| \| NM_001204384.1 \| \|  \| GCAGTAGCTGCGCTGATAGA \| \|  \| \|  \| \| GAPDH \| AGGGCTGCTTTTAACTCTGGT \| \| 206 \| \| NM_001289745.2 \| \|  \| CCCCACTTGATTTTGGAGGGA \| \|  \| \|  \| |
| --- | --- | --- | --- | --- | --- | --- | --- | --- | --- | --- | --- | --- | --- | --- | --- | --- | --- | --- | --- | --- | --- | --- | --- | --- | --- | --- | --- | --- | --- | --- | --- | --- | --- | --- | --- | --- | --- | --- | --- | --- | --- | --- | --- | --- | --- | --- | --- | --- | --- | --- | --- | --- | --- | --- | --- | --- | --- | --- | --- | --- | --- | --- | --- | --- | --- | --- | --- | --- | --- | --- | --- | --- | --- | --- | --- | --- | --- | --- | --- | --- | --- | --- | --- | --- | --- | --- | --- | --- | --- | --- | --- | --- | --- | --- | --- | --- | --- | --- | --- | --- | --- | --- | --- | --- | --- | --- | --- | --- | --- | --- | --- | --- | --- | --- | --- | --- | --- | --- | --- | --- | --- | --- | --- | --- | --- | --- | --- | --- | --- | --- | --- | --- | --- | --- | --- | --- | --- | --- | --- | --- | --- | --- | --- | --- | --- | --- | --- | --- | --- | --- | --- | --- | --- | --- | --- | --- | --- | --- | --- | --- | --- | --- | --- | --- | --- | --- | --- | --- | --- | --- | --- | --- | --- | --- | --- | --- | --- | --- | --- | --- | --- | --- | --- | --- | --- | --- | --- | --- | --- | --- | --- | --- | --- | --- | --- | --- | --- | --- | --- | --- | --- | --- | --- | --- | --- | --- | --- | --- | --- | --- | --- | --- | --- | --- | --- | --- | --- | --- | --- | --- | --- | --- | --- | --- | --- | --- | --- | --- | --- | --- | --- | --- | --- | --- | --- | --- | --- | --- | --- | --- | --- | --- | --- | --- | --- | --- | --- | --- | --- | --- | --- | --- | --- | --- | --- | --- | --- | --- |
